# Adapting Crops to Rising Temperatures: Understanding Heat Stress and Plant Resilience Mechanisms

**DOI:** 10.3390/ijms262110426

**Published:** 2025-10-27

**Authors:** Anand Kumar, Pandiyan Muthuramalingam, Reetesh Kumar, Savitri Tiwari, Laxmidas Verma, Sujeong Park, Hyunsuk Shin

**Affiliations:** 1 Faculty of Agricultural Sciences, GLA University, Mathura 281406, Uttar Pradesh, India; 2 Department of GreenBio Science, College of Agriculture and Life Sciences, Gyeongsang National University, Jinju 52828, Republic of Korea; sjp@gnu.ac.kr (S.P.); shinpomo@gnu.ac.kr (H.S.); 3 Department of Biotechnology and Bioengineering, School of Biosciences & Technology, Galgotias University, Greater Noida 201310, Gautam Buddha Nagar, India; reetesh.kumar@galgotiasuniversity.edu.in; 4 Department of Life Sciences, School of Biosciences & Technology, Galgotias University, Greater Noida 201310, Gautam Buddha Nagar, India; savitri.tiwari2985@gmail.com; 5 Department of Genetics and Plant Breeding, Acharya Narendra Deva University of Agriculture and Technology, Kumarganj, Ayodhya 224229, Uttar Pradesh, India; laxmidasverma8@gmail.com

**Keywords:** heat stress, physiological responses, molecular responses, epigenetic modifications

## Abstract

Global temperature rise has become a critical challenge to agricultural sustainability, severely affecting crop growth, productivity, and survival. Human-induced climate change and greenhouse gas emissions cause heat stress, disrupting plant metabolism and physiology at all developmental stages from germination to harvest. Elevated temperatures during germination impair water uptake, enzyme activity, and energy metabolism, leading to poor or uneven seedling emergence. At key phases such as flowering and grain filling, heat stress limits photosynthesis and transpiration by inducing stomatal closure, restricting carbon dioxide intake, and reducing photosynthetic efficiency. The reproductive stage is particularly vulnerable to high temperatures, impairing pollen viability, preventing anther dehiscence, and reducing fertilization success. Membrane instability further accelerates chlorophyll degradation and leaf senescence. Heat stress also alters biochemical and hormonal balances by disrupting the synthesis and signaling of auxins, gibberellins, and abscisic acid (ABA). Elevated ABA promotes stomatal closure to enhance stress tolerance, while increased ethylene levels trigger premature leaf senescence and abscission. These hormonal shifts and oxidative stress hinder plant growth and reproduction, threatening global food security. Although plants employ adaptive mechanisms such as heat shock protein expression and stress-responsive gene regulation, current strategies remain inadequate, highlighting the urgent need for innovative approaches to improve crop resilience under rising temperatures.

## 1. Introduction

Heat stress is a major challenge for agriculture because it can cause irreversible damage to crop plants [1]. Several key factors contribute to rising heat stress, including greenhouse gases, chlorofluorocarbons, human activities, and elevated CO_2_ levels. Numerous predictive models have been developed to assess the impact of climate change on crop yields [2]. Many studies show that increasing temperatures alone could reduce global yields of maize by 7.4%, wheat by 6.0%, soybean by 3.1% and rice by 3.2% [3]. In addition, projections indicate that the frequency and duration of extreme heat events may rise by about 50% by 2050 and 90% by 2100, leading to substantial yield losses in crops [4]. Warm season crops such as cucumber, cowpea and cotton exhibit greater tolerance to high temperatures, whereas cool-season crops like lentil and wheat experience reduced germination when soil temperatures exceed 24–26 °C [5]. Rising temperatures induce heat stress, altering crop plants on morphological, physiological and molecular processes. In severe cases, heat stress can kill cells within minutes or even destroy entire plants. The damage largely results from disrupted photosynthesis and respiration, accumulation of misfolded proteins and excessive reactive oxygen species (ROS) [6]. Young seedlings are especially vulnerable to heat stress due to their small size, proximity to hot soil and shallow roots, which hinder water retention. Moreover, reproductive tissues active during flowering and gametogenesis are also highly sensitive, often causing reduced fertility, poor seed set and significant yield losses [7]. Among all factors, the timing and duration of heat stress most strongly affect plant growth. Significant progress has been made in understanding how crops and model plants respond to moderately high temperatures or acute heat stress [8]. However, in natural environments, plants are frequently subjected to multiple and recurring episodes of heat stress rather than a single event. However, plants possess the ability to activate stress-tolerance mechanisms continuously. In addition, a sustained or prolonged response can lead to growth inhibition and a reduction in overall fitness. Upon the cessation of a heat event, certain stress-induced changes are reversed, while others are retained as adaptive modifications that enable plants to respond more rapidly and efficiently to subsequent stress exposures [9]. These adaptive traits persist over time, referred to as stress memory. Recent studies show that stress memory and resetting are actively regulated [10]. Understanding the balance between retaining and resetting these responses is critical for breeding crop varieties that survive and thrive under increasingly unpredictable and extreme climate conditions. In this context, exploring how heat stress memory (thermomemory) is regulated, how it is reset and which key mechanisms require further research remains a high priority [11].

Heat stress is especially damaging when it coincides with critical crop development stages, particularly reproduction. The reproductive phase is highly vulnerable, often causing significant yield losses [11]. Moreover, elevated temperatures increase biomolecular movement, disrupting plasma membrane stability by altering permeability and fluidity. This imbalance leads to the leakage of essential ions and amino acids [12]. A 5–10 °C rise above a plant’s optimal growth temperature can rapidly trigger ROS accumulation, causing severe oxidative damage, especially in photosystems I and II [13]. Heat stress also causes protein and lipid denaturation, mitochondrial dysfunction, and membrane degradation, collectively leading to cellular starvation, reduced ion flux and toxic metabolite buildup [14]. These stress induced disruptions set off a cascade of molecular, transcriptional, phenological and physiological changes that profoundly affect plant growth, development and survival. Insights into plant responses to systemic heat stress have been progressively revealed through studies in Arabidopsis [15]. In addition, heat shock factors (HSFs) and heat shock proteins (HSPs) are central to the heat shock response (HSR). However, signaling molecules such as calcium ions (Ca^2+^), nitric oxide (NO) and various phytohormones regulate HSF activity and activate HSR pathways [16]. The expression of HSR genes is further fine-tuned by noncoding RNAs (ncRNAs) and epigenetic modifications. A critical component of the heat stress response is the unfolded protein response (UPR) in the endoplasmic reticulum (ER), which helps alleviate proteotoxic stress [17]. Thermosensitive organelles, including mitochondria and chloroplasts also deploy adaptive strategies to withstand elevated temperatures. Different crop species vary in heat-tolerance thresholds, beyond which essential physiological processes are severely impaired [18]. This review provides an in-depth analysis of how heat stress affects plant physiology, emphasizing key cellular and molecular alterations under elevated temperatures. It explains how heat stress disrupts essential processes such as photosynthesis, respiration, and membrane stability, leading to oxidative damage and metabolic imbalance, while highlighting the roles of HSPs, antioxidants, and hormonal signaling pathways in limiting damage and maintaining homeostasis. The review further notes that these stress-induced responses vary across species and genotypes, reflecting considerable genetic diversity in heat tolerance, and underscores the importance of understanding such variation to assess how plants at different developmental stages from germination to reproduction cope with thermal extremes. However, reproductive phases such as flowering and grain filling are identified as particularly vulnerable, often resulting in significant reductions in yield and quality.

## 2. Plant Mechanisms and Responses During Heat Stress

**Figure 1:**
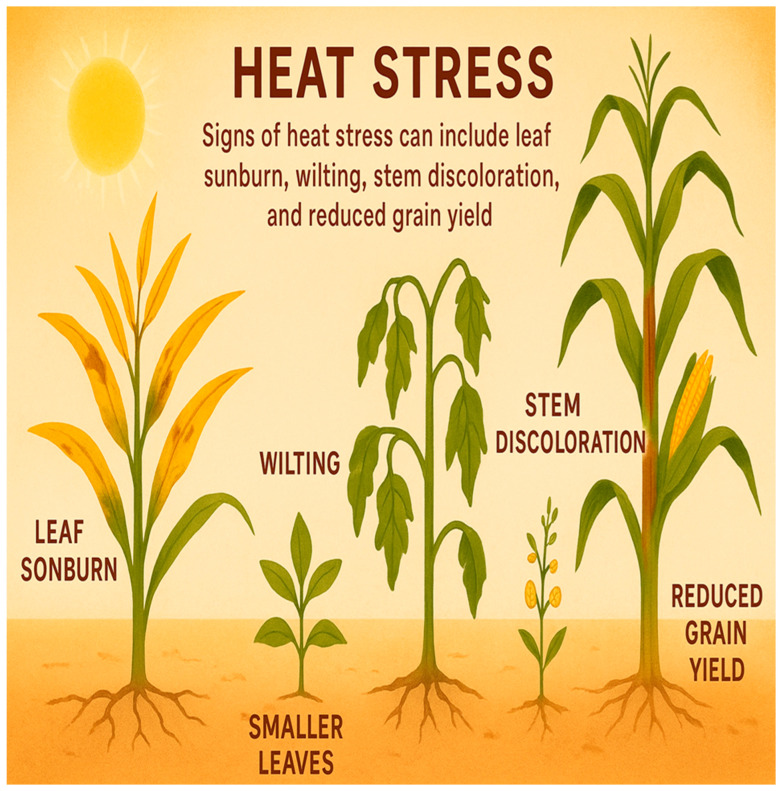
Schematic representation illustrating the effects of heat stress on plant growth.

Heat stress represents a major challenge for plants, significantly constraining their growth, development, metabolism and overall productivity. The resulting plant response alterations can exert positive and negative effects (Figure 1) on various morphological, physiological, hormonal and biochemical processes.

### 2.1. Plant Morphological Responses to Stress

Heat stress poses a significant challenge in tropical climates, severely restricting crop growth, development and productivity. Its detrimental effects are observed across a wide range of crops, including wheat, rice, maize, pearl millet, sorghum, barley, Brachypodium, Arabidopsis, pea and tomato. Heat stress leads to irreversible pre- and post-harvest losses, evident as leaf sunburn, scorching of leaves, stems, shoots and twigs, inhibited root development, fruit discoloration and substantial yield reductions. Prolonged exposure to high temperatures can alter leaves’ size, shape and orientation, often the first organs to show visible signs of heat stress [19]. New leaves tend to be smaller with reduced surface area to minimize heat absorption. In addition, some plants display paraheliotropism, reorienting leaves vertically to avoid direct sunlight during peak heat hours. Although these adaptations help reduce thermal load, they can limit light capture, ultimately reducing photosynthesis and biomass accumulation [20]. Moreover, wilting is one of the most prominent symptoms of heat stress, occurring when internal cell turgor pressure declines due to excessive water loss through transpiration. Under conditions of high temperature and low relative humidity, plants experience accelerated water loss, particularly when stomata remain open [21,22]. To counter this, many plants, especially monocots such as rice and wheat, exhibit leaf rolling, which reduces the surface area exposed to sunlight and conserves moisture. Other common symptoms include leaf scorching on sun exposed tissues and chlorosis, characterized by leaf yellowing caused by chlorophyll breakdown and chloroplast damage [23]. Heat stress significantly affects shoot growth and stem development, often causing stunted growth. This results primarily from inhibited cell elongation and expansion, and reduced meristematic activity, leading to shorter internodes and decreased overall plant height. In cereal crops such as maize and sorghum, stem elongation is particularly sensitive to elevated temperatures during vegetative and early reproductive stages [24,25].

Heat stress compromises stem structural integrity and development by damaging membranes and cell walls and inducing oxidative stress, which can produce cracking, swelling and reduced stem girth that disrupt vascular continuity and impede water and nutrient transport. In addition, heat-driven increases in ROS and light stress induce anthocyanin biosynthesis in stems, providing photoprotection and explaining observed changes in stem coloration [26]. At the meristematic level, elevated temperature perturbs hormonal homeostasis and limits carbon availability, thereby inhibiting the formation and expansion of the first and subsequent nodes. In sugarcane, these combined effects manifest as smaller nodes and altered apical dominance that promote increased tillering but reduce internode elongation and overall biomass accumulation, consistent with previously reported reductions in total yield under recurrent heat stress [27]. Though less visible, the root system undergoes profound morphological changes under heat stress. Elevated soil temperatures restrict root growth, shorten root length and reduce overall root biomass [28]. In many species, excessive heat causes root shrinkage and fewer root hairs, severely limiting water and nutrient uptake. Root elongation is especially temperature-sensitive, as high temperatures inhibit cell division and expansion in the root apical meristem [29,30]. Elevated soil temperatures can also induce root browning and necrosis, especially under dry conditions, thereby exacerbating the detrimental effects of heat stress. Moreover, shallow root development is common in plants exposed to extreme heat, especially those that prematurely halt growth as an avoidance strategy. In contrast, specific heat-resilient cultivars develop deeper or more extensive root systems, allowing access to moisture rich soil layers and improving drought avoidance and heat tolerance [31]. Heat stress also reduces total biomass production, indirectly affecting traits linked to growth and yield.

Even though morphological responses to heat stress appear across all developmental stages, from vegetative to reproductive phases. Seed germination and seedling vigor are particularly vulnerable and severe stress can lead to plant mortality. For example, in maize, coleoptile growth declines sharply at 40 °C and stops entirely at 45 °C [32]. In addition, heat stress poses a major threat to agricultural productivity by disrupting a wide range of physiological and morphological processes. Understanding these impacts is crucial for developing strategies to mitigate heat stress and enhance crop resilience amid rising global temperatures [33,34]. The optimum temperature for wheat flowering (anthesis) and grain filling ranges from 12 °C to 22 °C, respectively. Exposure to higher temperatures during these stages reduces grain number and size and during maturation, increased heat stress further decreases the number of grains per spike [35]. These findings underscore the substantial impact of heat stress on plant morphological productivity. Disruption of key processes such as shoot growth and node formation severely impairs overall plant development and biomass accumulation, ultimately leading to yield losses and posing a serious challenge to global food security. Therefore, understanding these effects and developing effective mitigation strategies are critical for sustaining agriculture in the face of climate change [36].

### 2.2. Physiological Adaptation of Plants to Heat Stress

Heat stress induces irreversible alterations in plant physiological processes, often resulting in considerable yield losses. Breeding and developing heat-tolerant cultivars represents one of the most effective approaches to counter these adverse effects. Crucial physiological traits such as stomatal regulation, cell membrane stability, canopy temperature, and chlorophyll content are reliable markers for assessing plant responses to elevated temperatures.

#### 2.2.1. Stomatal Conductance Activity During Heat Stress

Stomatal conductance which measures the rate of CO_2_ uptake and water vapor loss through the stomata, serves as a key indicator of plant responses to heat stress [37,38]. In addition, mild heat stress can trigger stomatal opening to enhance transpiration and cool leaves [39].However, prolonged or intense stress typically causes stomata to close to minimize water loss, especially during concurrent drought conditions. This process is mediated by the accumulation of abscisic acid (ABA) in leaves, which triggers signaling pathways in guard cells. ABA induces ion efflux (K^+^, Cl^−^ and malate) from guard cells, reducing their turgor pressure and causing stomatal pores to close [40]. In addition, regulation of stomatal conductance is a complex process mediated by signaling molecules such as hydrogen peroxide (H_2_O_2_), nitric oxide (NO), and calcium ions (Ca^2+^), which work together to control stomatal opening and closing [41]. Moreover, heat can also induce oxidative stress that disrupts turgor pressure in guard cells, altering stomatal responsiveness. The impact of heat stress on stomatal conductance varies among crops and is influenced by growth developmental stages, water availability and genetic makeup. A key adaptive mechanism by which plants alleviate heat stress is evaporative cooling, wherein the loss of water vapor through open stomata dissipates excess heat effectively lowering leaf temperature and stabilizing the microclimate around the foliage [42]. Daytime stomatal conductance facilitates this evaporation and helps maintain optimal leaf temperature. Under extreme heat conditions, stomatal conductance often declines as a consequence of both biochemical and physiological constraints [43,44,45]. High temperatures accelerate the denaturation and degradation of Rubisco, the key enzyme responsible for CO_2_ fixation in the Calvin cycle [46]. The resulting reduction in Rubisco content and activity diminishes the demand for internal CO_2_, which signals the stomata to close to maintain internal homeostasis [47]. Thus, stomata play a central role in regulating transpiration and leaf cooling. A wider stomatal aperture enhances transpiration and photosynthetic activity by promoting the diffusion of water vapor and carbon dioxide. Numerous studies highlight stomatal conductance and photosynthetic efficiency as valuable indicators for detecting and assessing plant heat stress [48].

#### 2.2.2. Cell Membrane Thermostability During Heat Stress

Sullivan (1974) introduced a protocol to evaluate cell membrane thermostability, a critical measure of heat tolerance [49]. Cell membranes are essential for maintaining cellular integrity, facilitating transport and regulating responses to environmental stimuli. Under normal conditions, the semi-fluid structure, mainly composed of phospholipids, proteins and sterols, allows membranes to remain flexible and dynamic, enabling selective transport of molecules and proper positioning of enzymes and receptors [50]. This structure also facilitates efficient signal transduction, cellular communication, shape changes and energy transduction, supporting essential physiological processes [51]. When temperatures rise above the optimal range, heat stress threatens membrane stability. The plasma membrane is often the first cellular structure affected, and its stability largely determines plant survival, growth and productivity, especially in sessile organisms that are directly exposed to environmental extremes [52,53].

Heat stress induces profound alterations in membrane structure and function. One of the earliest effects is increased membrane fluidity, as elevated temperatures disrupt the lipid bilayer and reduce its structural integrity. This excessive fluidity impairs membrane associated proteins, disrupts ion gradients, hinders nutrient transport, and interferes with signal transduction pathways [54]. Heat stress also accelerates the generation of ROS, which trigger lipid peroxidation and oxidative degradation of membrane lipids that produce malondialdehyde (MDA) and other cytotoxic compounds [55,56]. Lipid peroxidation further weakens the membrane, increasing permeability and causing leakage of cellular contents, which can lead to cell death if damage is severe.

In addition, high temperature disrupts the lipid bilayer and destabilizes membrane proteins, including transporters, receptors and enzymes, often causing their unfolding or aggregation, further impairs membrane functions [57]. Heat stress can additionally disturb membrane asymmetry, the uneven distribution of lipids between inner and outer leaflets, resulting in abnormal signaling and compromised cell survival [58]. Despite these challenges, cells have evolved adaptive mechanisms to maintain membrane stability under heat stress. A key strategy is altering membrane lipid composition, particularly by increasing the proportion of saturated fatty acids, which pack tightly and reduce fluidity, thereby preserving membrane integrity at higher temperatures [59,60]. Additionally, accumulating specific sterols in plant cells enhances membrane rigidity and resilience. Another key component of the heat stress response is the production of HSPs. These molecular chaperones refold denatured proteins, prevent protein aggregation, and, in some cases, directly stabilize membrane structures. Small heat shock proteins (sHSPs) can associate with membranes and protect them from heat-induced damage by acting as molecular shields [61].

Moreover, activation of antioxidant defense systems is also vital for limiting membrane oxidative damage. Enzymatic antioxidants, including superoxide dismutase (SOD), catalase (CAT), and ascorbate peroxidase (APX), play a crucial role in scavenging ROS and preventing lipid peroxidation. Meanwhile, non-enzymatic antioxidants such as tocopherols (vitamin E), ascorbic acid (vitamin C) and glutathione protect membrane lipids from oxidative damage, thereby preserving membrane integrity [62]. In addition, osmoprotectants, or compatible solutes, provide another layer of protection. Compounds such as proline, glycine betaine and trehalose accumulate under stress, where they stabilize proteins and lipids and help maintain cellular osmotic balance [63]. These molecules preserve membrane integrity by protecting the hydration shell around membrane components and reducing thermal denaturation. In addition, the cytoskeleton composed of actin filaments and microtubules interacts with the plasma membrane to provide mechanical support. During heat stress, cytoskeletal reorganization reinforces membrane stability and helps maintain cell shape and function [64]. On the other hand, electrolyte leakage assays detect ion loss from heat-stressed tissues, with higher leakage indicating greater membrane damage, while lipid peroxidation assays measure MDA accumulation as an indicator of oxidative injury [65]. Cell membrane stability (CMS) strongly correlates with yield stability under heat stress in plants. Varieties with higher CMS typically show better photosynthetic efficiency, reproductive success and biomass production. Consequently, CMS has become a key selection criterion in breeding programs aimed at developing heat-tolerant cultivars of crops such as wheat, rice, maize and soybean, which are highly susceptible to temperature extremes during critical growth stages [66].

Maintaining membrane stability under heat stress is a complex, dynamic process that integrates structural adjustments, molecular chaperoning, antioxidant protection, osmotic regulation and cytoskeletal support. Together, these defenses safeguard cellular functions in hostile thermal environments. With climate change driving more frequent and intense heat waves, understanding the mechanisms of CMS has become critical for agriculture [67,68]. Future research should focus on identifying key regulatory genes, developing bioengineered crops with enhanced CMS and exploring novel chemical protectants to improve resilience to rising global temperatures [69]. Moreover, cell thermostability, a key physiological trait controlled by relatively few genes, is widely used to identify heat-tolerant genotypes. It has been successfully applied in soybean [70], potato [71], sorghum [72], barley [73], tomato [74] and rice [75]. A standard method to assess cell membrane thermostability involves measuring electrolyte leakage from leaf disks exposed to heat stress, with higher leakage indicating greater membrane damage and lower stability. Additionally, chlorophyll fluorescence and membrane leakage assays provide sensitive measures of physiological responses to high temperatures, as demonstrated in cotton [76].

Canopy temperature is another important parameter for assessing plant heat stress. Measured with infrared thermometry, it identifies heat-tolerant genotypes across crops [65]. Photosynthesis is particularly sensitive to elevated temperatures in both C3 and C4 plants [77]. In C3 species, the photosynthetic rate depends heavily on the concentration of CO_2_ within intercellular leaf spaces. Heat stress disrupts both the light-dependent reactions in thylakoid membranes and carbon assimilation in the chloroplast stroma, which are the primary sites of thermal damage [78]. Reduced photosynthetic efficiency limits carbohydrate supply to developing tissues, ultimately hindering growth and yield. Heat stress significantly decreases photosynthesis and chlorophyll content, with a pronounced decline in the chlorophyll a:b ratio, especially in newly developed leaves [79]. These reductions are often accompanied by increased ROS production, further impairing photosynthetic performance. Heat stress also reduces leaf area and water potential, further exacerbating the decline in photosynthetic activity. Under elevated temperatures, photosynthesis may shift from non-cyclic to cyclic photophosphorylation. This transition, along with electron transport chain disruption, degradation of essential proteins and pigment loss, severely compromises efficiency [80]. High temperatures can damage Photosystem II within the thylakoid membranes during the vegetative stage, causing membrane instability and further loss of photosynthetic capacity [81]. Changes in photosynthetic and transpiration rates are primary indicators of heat stress in plants. Elevated temperatures cause ultrastructural damage within chloroplasts, including thylakoid membrane destabilization, grana swelling and disruption of carbon metabolism in the stroma processes essential for growth and development [82,83]. Heat stress impairs both Photosystem I (PSI) and Photosystem II (PSII), leading to photoinhibition and reduced photosynthetic efficiency [84,85].

Under moderate heat stress, plants may maintain balanced electron flow or activate protective mechanisms against excess excitation energy, especially under fluctuating light. It is demonstrated that PSI photoinhibition in tobacco occurred at 25 °C and 42 °C when exposed to fluctuating light. In such cases, PSII transfers electrons to PSI, which becomes photodamaged when electron sinks cannot efficiently dissipate the surplus electrons [86]. Photoinhibition occurs when the photosystems, PSI or PSII, are damaged or inactivated by excess light or stress conditions, reducing their ability to transfer electrons efficiently. This limits ATP and NADPH production, thereby constraining the Calvin–Benson cycle and CO_2_ assimilation [85]. In C4 plants, the higher CO_2_ fixation per leaf area demands greater electron transport capacity, making them particularly sensitive to limitations in photosystem efficiency [85,86,87]. Yan et al. (2013) also reported significant PSII inactivation in sorghum under high-temperature stress [88].

Moreover, respiration is essential for supporting photosynthesis by supplying the energy and carbon skeletons required for metabolic processes. Inhibition of respiratory activity reduces energy production, thereby exacerbating photoinhibition under stress conditions [89,90,91]. Heat stress also disrupts mitochondrial membrane integrity, impairing oxidative phosphorylation and respiratory efficiency. Many chloroplast proteins are encoded by the nuclear genome, and heat stress induced damage to the nuclear envelope can hinder their transport. This disruption impairs photoprotection delays the repair of damaged photosystems and exacerbates structural damage to the photosynthetic machinery [92]. Additionally, respiration rates often rise sharply at 40–50 °C, increasing respiratory carbon losses, reducing ATP production, and elevating ROS, disturbing cellular energy balance and metabolism [93]. Heat also affects the kinetics of Rubisco and the solubility of CO_2_. Rubisco, the key enzyme in photosynthesis and photorespiration, has dual carboxylase and oxygenase activities [94]. High temperatures favor its oxygenase activity, increasing photorespiration and reducing photosynthetic efficiency. Meanwhile, the concentration of CO_2_ within mesophyll cells becomes limiting for carboxylase activity, and increased CO_2_ losses through photorespiration further lower net photosynthetic rates under heat stress [95].

### 2.3. Plant Hormonal Responses to Heat Stress

Several plant hormones play pivotal roles in maintaining physiological functions under heat stress. Hormones such as ABA, salicylic acid (SA), and ethylene typically increase in response to high temperatures, whereas auxins, cytokinins, and gibberellins often decline [96]. Among these, ABA is a key mediator of abiotic stress responses especially for heat stress. It helps plants tolerate elevated temperatures by promoting stomatal closure through osmotic adjustments, thereby reducing water loss. ABA also regulates the expression of numerous heat-responsive genes, enhancing stress tolerance at the molecular level [97]. In addition, ABA modulates ROS levels, particularly in guard cells, by regulating NADPH oxidases such as Respiratory Burst Oxidase Homolog (Rboh) proteins [98]. This dual role in controlling stomatal behavior and ROS signaling highlights ABA’s central importance in improving plant resilience to heat stress.

A study examining the effects of high-temperature stress on tassel development in maize selected two contrasting genotypes: the heat-tolerant Zhengdan 958 (ZD958) and the heat-sensitive Xianyu 335 (XY335) [99,100]. Exposure to elevated temperatures during tassel development significantly reduced tassel size and the area available for anther dehiscence, severely hindering pollen dispersal and resulting in a marked decline in pollen production. Heat stress also adversely affected pollen development, producing malformed grains with reduced viability and significantly lower germination rates. Biochemical assays showed a marked increase in the activity of key ROS scavenging enzymes, including SOD, POD, and CAT [101,102]. In addition, enhanced activities of APX and GR were observed, indicating an upregulation of the antioxidant defense system [102]. Concurrently, levels of MDA and H_2_O_2_ rose substantially, with the heat-sensitive XY335 exhibiting more severe oxidative damage than the resilient ZD958 [103]. These changes help mitigate oxidative damage to cellular membranes, proteins, and nucleic acids, thereby improving stress tolerance in the heat-tolerant genotype.

High temperatures further disrupted the hormonal balance in tassels. Both genotypes exhibited significant reductions in zeatin and SA, accompanied by increases in ABA and gibberellic acid (GA). In addition, responses of jasmonic acid (JA) and indole-3-acetic acid (IAA) were genotype specific. In the heat-tolerant ZD958, both JA and IAA increased under heat stress, whereas in the heat-sensitive XY335, their levels declined [104]. These ROS metabolism and hormonal regulation shifts collectively disrupted tassel development, culminating in substantial reductions in pollen quantity, viability, and germination potential [105]. Interestingly, the bZIP transcription factor TRITD5Av1G026510 was markedly downregulated under stress conditions [106]. bZIP (basic leucine zipper) proteins are involved in a wide range of plant physiological and developmental processes, including hormonal signaling, light responses, photomorphogenesis, seed germination and maturation, as well as floral induction and flower development [105,106]. Notably, the *A. thaliana* ortholog, bZIP10 (AT4G02640), has been shown to activate HSP90 transcription, particularly under elevated glutathione levels in stressed leaf tissues [107,108]. This observation suggests that the wheat bZIP homolog may similarly function in stress-responsive signaling pathways, potentially through redox-regulated transcriptional control [105,108]. Notably, an ortholog of this gene in *A. thaliana*, bZIP10 (AT4G02640), has been implicated in the transcriptional activation of HSP90, particularly under elevated glutathione conditions in stressed leaf tissues. This suggests that the wheat bZIP homolog may similarly participate in stress-responsive signaling networks, potentially through redox-regulated transcriptional control [108,109]. In addition, cytokinin, a key phytohormone, is crucial role in regulating multiple aspects of plant growth and development. Although its developmental functions are well established, its involvement in abiotic stress tolerance remains complex and, at times, contradictory [110], due to intricate cross-talk between cytokinin signaling and stress-responsive pathways [111]. Moreover, cytokinin often exhibits antagonistic interactions with GAs at different developmental stages. For instance, it can inhibit GA-dependent processes such as hypocotyl elongation and leaf serration in tomato, underscoring the dynamic balance between cytokinin and GA as a critical regulatory mechanism, particularly under stress conditions where the allocation of resources between growth and defense is essential [112]. This antagonistic interaction between cytokinin and GA arises from their contrasting roles in regulating growth and stress responses. Cytokinin generally promotes cell division and differentiation, while GA stimulates cell elongation and organ expansion [113]. Under stress conditions, elevated cytokinin levels can suppress GA biosynthesis or signaling, thereby reducing GA-dependent processes such as hypocotyl elongation and leaf serration [114]. This shift favors resource allocation toward defense and stress adaptation rather than growth. The cytokinin and GA cross-talk thus act as a regulatory mechanism that enables plants to modulate developmental plasticity and maintain energy homeostasis under adverse environmental conditions [115,116].

Additionally, GAs are a diverse group of naturally occurring diterpenoids that regulate key developmental processes, including seed germination, stem elongation, flowering and fruit development. A primary mechanism by which GAs promote growth is through the degradation of DELLA proteins, which act as negative regulators of GA signaling [117]. Beyond their developmental roles, GAs are critical for plant responses to heat stress. For example, in *A. thaliana*, acute heat stress (50 °C for 3 h) severely inhibits seed germination and seedling growth. In contrast, exogenous application of GA_3_ (50 μM) alleviates these effects, enhancing germination and early seedling growth under high-temperature conditions [118]. Notably, GA function under heat stress involves cross-talk with SA pathways, modulating seed germination and seedling development by influencing both SA biosynthesis and signaling. Enhancing GA activity, both through GA_3_ application or overexpression of GASA (Gibberellin-regulated protein) genes, increases SA levels and improves thermo tolerance in Arabidopsis, highlighting the integrative role of GA in coordinating stress adaptation [119]. Under heat stress, the interaction between GA and SA reflects the intricate hormonal cross-talk that coordinates growth and defense responses. GA enhances SA biosynthesis by upregulating key enzymes such as isochorismate synthase 1 (ICS1) and promotes SA signaling components involved in thermotolerance [120]. This interaction helps activate heat-responsive transcription factors and antioxidant defense systems, thereby improving cellular protection and repair mechanisms during thermal stress [121]. Moreover, GA-induced expression of GASA genes contributes to maintaining redox homeostasis and stabilizing membrane integrity [122]. Consequently, the enhancement of GA activity not only promotes developmental recovery but also strengthens the SA-mediated defense network [123]. In addition, rapid elongation of stems and hypocotyls is a classic morphological adaptation to elevated temperatures, tightly regulated by GA. Under heat stress, reduced GA biosynthesis alters hypocotyl elongation, highlighting the importance of dynamic GA regulation for temperature-responsive growth [124]. This GA-mediated elongation acts synergistically with auxin signaling pathways, while the brassinosteroid (BR) pathway becomes increasingly important as plants progress through different developmental stages. GA signaling enhances the post-translational activity of PHYTOCHROME INTERACTING FACTOR 4 (PIF4) under elevated temperatures, reinforcing GA’s central role in coordinating growth responses during thermal stress [125].

The timing of flowering is a critical developmental milestone with major implications for reproductive success and pollinator synchronization. Elevated temperatures often accelerate flowering, although the magnitude of this response varies across species. Under heat stress, PIF4 plays a central role by activating FLOWERING LOCUS T (FT), a key integrator of floral induction. PIF4 activity is enhanced by DELLA protein degradation, a process promoted by GA. Thus, rising GA levels under high temperatures facilitate FT expression via PIF4, advancing the flowering phase [126]. In addition, heat stress significantly alters the hormonal balance in plants, affecting growth, development and yield. To investigate the role of hormonal priming in stress mitigation, a study evaluated the effects of ABA seed priming (10^−6^ M) on growth and cytokinin dynamics in two closely related wheat species namely, *Triticum aestivum* (‘Podolyanka’) and *Triticum spelta* (‘Frankenkorn’). Seeds were primed with either water (control, C-plants) or ABA solution (ABA^+^ plants) [127]. During heat stress, shoot biomass decreased in ABA treated *T. aestivum*, whereas root biomass increased in ABA treated *T. spelta*, indicating species-specific responses to ABA priming [128]. Following the recovery period, ABA treated wheat plants still exhibited reduced shoot biomass compared to controls, while ABA treated spelt plants outperformed their control counterparts, suggesting a more robust adaptive response in *T. spelta* [129]. During heat stress, ABA priming triggers species-specific physiological and developmental responses. In *T. aestivum*, ABA likely prioritized stress defense mechanisms over shoot growth, such as stomatal closure to reduce transpiration and activation of stress-responsive genes, which resulted in reduced shoot biomass [127,128,129,130,131]. In contrast, in *T. spelta*, ABA enhanced root growth, likely by improving hydraulic conductivity and osmotic adjustment, enabling better water uptake and sustained metabolism under heat stress [127,128,129,132]. Moreover, hormonal profiling revealed a substantial increase in cytokinin levels in both shoots and roots of ABA-treated wheat plants under heat stress, rising by 76.8% and 313.3%, respectively, compared to non-stressed ABA plants [133]. In shoots, trans-zeatin-O-glucoside and isopentenyladenine increased 2.8-fold and 2.6-fold, respectively [134], while in roots, trans-zeatin and isopentenyladenine rose 2.8-fold and 23.3-fold, respectively [134]. This pattern indicates a differential regulation of cytokinin metabolism between shoots and roots with roots likely serving as the principal site of cytokinin synthesis especially for isopentenyl type cytokinins. Such regulation supports effective root-to-shoot signaling, thereby facilitating coordinated plant growth and enhancing stress adaptation [135]. These findings highlighted the critical role of ABA pre-treatment in modulating cytokinin biosynthesis and distribution, contributing to differential biomass allocation and enhanced stress resilience, particularly in *T. spelta* [136]. By 21 days post-recovery, the total cytokinin content in ABA-treated spelt plants remained markedly lower as 2.6-fold and 2.1-fold less than that of the non-stressed ABA and control groups, respectively [137]. In summary, ABA seed priming induced distinct shifts in cytokinin dynamics in winter wheat (*T. aestivum*, Podolyanka) and spelt (*T. spelta*, Frankenkorn) under heat stress [138]. In Podolyanka wheat, exogenous ABA enhanced cytokinin accumulation in both shoots and roots during and after heat exposure. In contrast, Frankenkorn spelt showed a decline in shoot cytokinin but a notable increase in root cytokinin during stress [139]. Even after recovery, the hormonal effects of ABA priming persisted, with elevated shoot but reduced root cytokinin in wheat, whereas both shoot and root cytokinin levels remained suppressed in spelt [140]. The reduced cytokinin levels in wheat roots could indicate a strategic reallocation of hormonal resources toward the shoots, where recovery processes are metabolically more demanding [141]. On the other hand, Spelt, being an ancient wheat species, is often characterized by slower metabolic reactivation and higher sensitivity to hormonal feedback regulation. ABA priming may have induced a stronger or prolonged inhibitory effect on cytokinin biosynthesis genes or enhanced cytokinin degradation via CKX activity [142].

Ethylene, a gaseous plant hormone, plays a key role in regulating growth and development, including seed germination, fruit ripening and responses to abiotic stresses [143]. Its role during heat stress is complex and species specific. For instance, in soybean, exposure to 40 °C enhances hypocotyl elongation, suggesting a positive regulatory role of ethylene under moderate heat. In contrast, the same temperature inhibits ethylene production in wheat leaves, indicating differential species responses to thermal stress [144]. These contrasting effects highlight the nuanced and context-dependent nature of ethylene signaling under elevated temperatures. SA also plays a significant role in the HSR by mitigating oxidative damage through detoxification of superoxide radicals, thereby protecting cellular membranes [145]. SA enhances thermotolerance by regulating HSP gene expression and activating antioxidant defense mechanisms, ultimately improving plant fertility and yield under heat stress [146]. In contrast to ABA, hormones such as gibberellins and cytokinins are generally downregulated under high temperatures, with declines associated with inhibited root and shoot growth and reduced dry matter accumulation [147]. This hormonal decline slows cell division and elongation, suppressing root and shoot growth. Additionally, increased activity of catabolic enzymes like GA2-oxidases and CKXs further reduces active hormone levels. [148]. Additionally, elevated temperatures reduce endogenous auxin levels, particularly in reproductive tissues such as anthers, which can negatively impact fertility and reproductive success [149].

### 2.4. Plant Reproductive Responses to Heat Stress

Plants generally perform optimally under favorable environmental conditions, but their survival declines markedly under heat stress. Without effective adaptation or mitigation mechanisms, they struggle to withstand adverse conditions. In response, plants modify their physiological and biochemical processes to enhance survival and maintain yield potential [150]. Heat stress during the reproductive phase is particularly damaging, as it disrupts critical processes such as inflorescence development, sporogenesis, gametogenesis, anthesis, pollination and fertilization [151] (Table 1). Each stage is susceptible to elevated temperatures, and impairment can severely compromise reproductive success, ultimately reducing crop yield.

In cereals, each inflorescence consists of multiple spikelets, each containing one or more florets, which serve as the basic reproductive units [183]. During the transition to the reproductive phase, the inflorescence meristem first differentiates into spikelet meristems, which subsequently give rise to floret meristems [184]. Each spikelet is subtended by two glumes that enclose one or more florets. For instance, in maize, each spikelet contains two florets enclosed by paired glumes, whereas wheat spikelets may bear several florets, and in rice, the glumes are typically reduced in size. Each floret comprises a lemma, palea, lodicules, stamens and carpels [185]. However, cereal grain yield largely depends on the number of fertile florets formed before anthesis, influenced by genetics and environment [186]. Spikelet development is a complex process involving the florets coordinated initiation and maturation, often occurring in parallel with stem elongation [187].

Following vegetative development, the ontogeny of floral structures begins with the initiation of floral organ primordia within the spikelets, which are arranged along the central axis of the inflorescence or its lateral branches [188]. A seminal study by Coen and Meyerowitz (1991) introduced the highly conserved ABC model of floral development, based on analyses of floral homeotic mutants in *A. thaliana* and *Antirrhinum majus* [189].

For example, the ABC model proposes that three primary classes of *MADS-box* genes *AGAMOUS*, *DEFICIENS*, and *SEPALLATA* regulate floral organ identity through combinatorial expression patterns. These genes act in overlapping domains to specify the four concentric floral whorls as follows: from the outermost to the innermost, the first whorl develops into sepals, the second whorl develops into petals, the third into stamens, and the fourth into carpels [190]. Over the past three decades, the ABC model has been significantly refined and expanded. The inclusion of D-class genes accounted for the regulation of ovule identity and development within the carpel [191]. Furthermore, the identification of four *SEPALLATA* genes (*SEP1–SEP4*) demonstrated that, combined with A, B, C and D-class *MADS-box* genes, these factors collaboratively determine floral organ identity in *A. thaliana* [192]. Elevated temperatures during flowering exert a detrimental effect on flower development, often leading to reduced floral inflorescence. This reduction is primarily attributed to disruptions in the finely tuned expression of MADS-box genes, which are essential for the properly developing floral structures, including florets, sepals, petals, stamens, carpels and spikelets [193]. Perturbation in A-class gene expression can hinder sepal development which is a critical first step in floral organogenesis. In addition, coordinated activity of A and B class genes is required for petal specification, and imbalances under heat stress can result in petal suppression [194]. Heat stress may also impair stamen development, and C-class gene function, essential for pistil formation, can be compromised, leading to pistil abnormalities [195]. Heat stress can cause transcriptional downregulation, misexpression, or delayed activation of these genes, resulting in imbalances that suppress petal initiation and pistil formation [196]. Such disruptions reflect the broader impact of heat stress on gene regulatory networks controlling reproductive development, ultimately affecting floral architecture and fertility [197].

Paradoxically, exposure to supraoptimal temperatures can induce precocious floral initiation, likely due to increased sensitivity of meristematic cells to endogenous floral-inducing signals [198]. This accelerated floral transition is also hypothesized to involve alterations in phytochrome homeostasis, specifically an increased proportion of the 730 nm absorbing form relative to the 660 nm absorbing form. Elevated temperatures can delay flowering in long-day plant species, implicating the 730 nm absorbing phytochrome in floral promotion under certain photoperiod conditions [199]. However, experimental evidence shows that diurnal exposure to elevated temperatures at 36 °C during the day and 26 °C at night for five days significantly reduces floral fertility compared with optimal conditions (31 °C) [200]. Comparative studies in sorghum reveal that heat stress applied 10, 5 or 0 days before anthesis markedly diminishes floral fertility and even exposure 15 days prior to anthesis can compromise reproductive success. The developmental window spanning 10 to 5 days before anthesis appears particularly sensitive, during which floral organs are highly susceptible to heat-induced damage [201].

#### 2.4.1. Heat Stress Responses During Microsporogenesis/Megasporogenesis

Meiosis, the fundamental process of reproductive cell division, occurs in both pollen mother cells (PMCs) and megaspore mother cells (MMCs) which are located in the anthers and ovules, respectively [202]. These precursor cells arise from sporogenous cells through successive mitotic divisions in various plant species, including rice, wheat, barley, *Brachypodium distachyon*, maize, sorghum, pearl millet, *A. thaliana*, pea and tomato [203]. However, the EXCESS MICROSPOROCYTES1 or EXTRA SPOROGENOUS CELLS (EMS1/EXS) genes control a key regulatory pathway for tapetum differentiation. In the absence of functional EMS1/EXS, PMCs form without proper tapetal cell differentiation, leading to non-viable pollen [204]. Although EMS1/EXS genes are also expressed in the gynoecium, their precise role in ovule identity remains unclear. However, some studies suggested that EMS1/EXS is essential for tapetum differentiation which provides nutrients and signals to developing PMCs without it, pollen becomes non-viable. In the gynoecium, EMS1/EXS may have minor or redundant roles in ovule development, explaining why its disruption primarily affects male fertility [205].

Heat stress can profoundly disrupt microsporogenesis, causing a range of developmental abnormalities. MADS-box transcription factors, which orchestrate key plant developmental processes, often show altered expression or activity under elevated temperatures, thereby perturbing the regulatory networks that control anther primordium differentiation and proper PMC development [206]. Furthermore, the *ameiotic1* gene is highly heat-sensitive, essential for transitioning from mitosis to meiosis in crops such as maize and rice. Its disruption interferes with chromosomal synapsis and homologous recombination, leading to asynapsis, premature desynapsis and ultimately, complete arrest of meiotic progression [207,208].

Beyond genetic regulation, multiple cellular and molecular mechanisms contribute to the partial or complete inhibition of microsporogenesis and megasporogenesis under heat stress [209]. Notably, the activity of protein kinases, which are essential for initiating and regulating meiotic events, is susceptible to temperature fluctuations. Heat-induced disruptions in kinase signaling can derail the meiotic program, although the precise molecular mechanisms and downstream effects remain incompletely understood [210]. Heat stress can impair kinase signaling pathways that regulate meiosis, disrupting chromosome segregation, spindle formation, and cell-cycle progression, which leads to defective gamete development. However, the exact molecular targets and downstream cascades remain only partially characterized [210]. Nutrient availability also critically influences reproductive success. In wheat, microsporogenesis is particularly sensitive to boron deficiency because boron is essential for cell wall formation, membrane stability and metabolic processes during early microsporogenesis. Boron deficiency exhibits progressive developmental disruptions beginning from the premeiotic interphase and extending through the late tetrad stages, while the subsequent mitotic phases, characterized by starch accumulation in pollen, remain comparatively less affected [211]. Similarly, nitrogen availability is crucial for floret development, beginning with initiating the third floret primordium and influencing subsequent reproductive outcomes [212].

#### 2.4.2. Heat Stress Responses During Microgametogenesis/Megagametogenesis

Beyond structural considerations, recent advances in transcriptomic and mutational analyses have highlighted the critical roles of specific genes in germline development and pollen regulatory networks [213]. In the majority of angiosperms (>70%), the embryo sac or female gametophyte follows the characteristic Polygonum-type developmental pattern, ultimately forming seven cells of four distinct functional types [214]. During ovule development, the solitary functional megaspore, typically located at the chalazal or proximal end, undergoes enlargement and proceeds through two successive rounds of mitosis without cytokinesis [215]. This produces a four-nucleate syncytium, with two nuclei positioned at each pole. A third mitotic division, accompanied by phragmoplast formation and cell plate development between sister and non-sister nuclei, initiates cellularization. This process establishes individual gametophytic cells, each enclosed by a cell wall [216].

Although female reproductive organs are often considered more resilient than male organs, emerging evidence from various crop species indicates that female reproductive development is also vulnerable to heat stress. Heat stress adversely impacts multiple components of the female gametophyte, including the egg cell, synergid cells and the embryo sac [217]. It is due to the effect of heat stress as heat stress impairs ovule development by disrupting hormonal balance, downregulating key genes, inducing oxidative damage and limiting nutrient supply, collectively reducing ovule viability, fertilization, and seed set [218]. These effects often result in reduced ovule numbers and ovule abnormalities. Elevated temperatures can impair gametophyte expansion and disrupt cell division within both egg and synergid cells [219]. Beyond structural and developmental effects, heat stress also disturb pistil metabolism, causing declines in adenosine triphosphate (ATP) levels and total soluble carbohydrates. This happened due to affecting mitochondrial function and enzymatic activity, which reduces ATP synthesis. Simultaneously, it disrupts carbohydrate metabolism, limiting the accumulation of soluble sugars needed for energy and osmotic balance [220]. Such metabolic disruptions can induce irreversible physiological changes prior to pollination and adversely affect subsequent post-pollination processes [221]. Similarly, in pearl millet, female reproductive tissues have been reported to be more sensitive to heat stress than male gametophytes. Under high temperatures, pistils experience greater oxidative damage than pollen grains, as evidenced by altered antioxidant enzyme activities and elevated ROS levels [222].

### 2.5. Molecular Mechanism of Heats Stress Response

Plants deploy diverse molecular mechanisms to cope with heat stress, with transcriptional regulation serving as a central adaptive strategy. Transcription factors act as key regulators, coordinating the expression of downstream effector genes that mitigate stress effects [223]. Heat stress triggers extensive gene expression and protein synthesis alterations, enabling plants to adjust their physiology and metabolism to elevated temperatures. The following sections provide an overview of selected molecular responses involved in heat stress adaptation [224].

#### 2.5.1. Role of Heat Shock Proteins in Heat Stress Response

Extensive research has shown that heat stress activates complex transcriptional regulatory networks in plants, triggering a cascade of molecular events essential for cellular survival. A key aspect of this response is the de novo production of HSPs, which is highly conserved molecular chaperones essential for cellular protection and enhancing stress tolerance [225]. HSP expressions are tightly regulated both temporally and spatially, varying across developmental stages and tissues. The high conservation of HSPs across plant species underscores their fundamental roles in stress adaptation. Heat stress regulates gene expression through transcriptional control, mRNA stability, translation efficiency, and protein activity, collectively strengthening cellular resilience to high temperatures [226].

In wheat, the *TaHSFA6e* gene, encoding a heat shock transcription factor, undergoes alternative splicing under heat stress, producing two functional isoforms as *TaHSFA6e-II* and *TaHSFA6e-III*. Notably, *TaHSFA6e-III* exhibits a greater capacity to activate transcription of three downstream *TaHSP70* genes than *TaHSFA6e-II* [227]. This enhanced activity is attributed to a 14-amino-acid peptide at the C-terminus of *TaHSFA6e-III*, generated through alternative splicing and predicted to form an amphipathic helix [227,228], which can interact with other proteins or DNA, stabilizing the transcription factor or enhancing its binding affinity to heat shock elements (HSEs). Functional analyses indicate that knockout of either *TaHSFA6e* or *TaHSP70* genes increases heat sensitivity in wheat. Moreover, *TaHSP70* proteins localize to stress granules during heat stress and regulate granule disassembly, facilitating translation re-initiation during recovery [227]. Polysome profiling reveals that mRNA translational efficiency is reduced in *TaHSP70* mutants compared to wild-type plants during recovery [229].

Additionally, the expression of HSPs such as HSA32 and HSP70T-2 is induced by AtMBF1c, a key factor in heat tolerance [230]. Supporting its potential role in translation, affinity purification assays show that the archaeal MBF1 protein from *Sulfolobus solfataricus* associates with the 30S ribosomal subunit during translation [203]. However, the mechanisms by which MBF1c modulates translation under heat stress in plants remain unclear and warrant further investigation [231]. Recent studies also highlight the importance of the endoplasmic reticulum protein processing pathway in maize heat tolerance. Twenty-seven genes linked to this pathway were identified, most encoding small sHSPs such as HSP26, 17.4 kDa class I sHSP, 17.5 kDa class II sHSP, 22.0 kDa class IV sHSP, 23.6 kDa mitochondrial sHSP and class I HSP3, indicating that sHSPs are crucial for enabling maize kernels to withstand elevated temperatures [232].

HSEs, present within the promoter regions of HSP genes are recognized and bound by HSFs, initiating transcriptional activation [233]. The conserved interaction between HSEs and HSFs forms the foundation of the heat shock response, emphasizing its crucial role in plant stress adaptation. Rapid HSP induction, which differs among species, is precisely controlled by intricate networks of transcription factors and their regulatory genes [234]. In addition, HSPs primarily function as molecular chaperones, assisting in the refolding of misfolded or denatured proteins, preventing irreversible aggregation, and maintaining cellular proteostasis [235]. Their functional diversity extends beyond stress responses, protein maturation and developmental regulation. For instance, in rice, heat stress during grain filling upregulates genes involved in starch metabolism and storage protein synthesis [236]. In *A. thaliana*, a network of 21 transcription factors regulates HSP genes alongside other stress-responsive genes, enabling extensive transcriptional reprogramming under heat stress [237]. Additionally, ROS produced under stress act as signaling molecules, activating HSFs and amplifying the heat shock response. Under heat stress, ROS accumulate due to disrupted electron transport and metabolic imbalance. These ROS function as secondary messengers, triggering signaling cascades that activate HSFs [238]. Additionally, ROS can modify redox-sensitive cysteine residues or influence protein kinases and phosphatases that regulate HSF activity. Once activated, HSFs bind to HSEs in target gene promoters, enhancing transcription of HSPs. This ROS-mediated signaling amplifies the heat shock response, providing cellular protection by stabilizing proteins, maintaining membrane integrity, and enhancing stress tolerance [239].

Plant HSPs are classified into three major classes based on molecular weight: HSP70, HSP90, and sHSPs, ranging from 15 to 30 kDa. Under heat stress, HSP70 and HSP90 are typically upregulated ~10-fold, whereas sHSPs can increase expression up to 200-fold [240]. HSPs are localized to multiple subcellular compartments including chloroplasts, mitochondria, ribosomes and cell walls reflecting their diverse and compartment specific functions [241]. For example, upon exposure to 42 °C, maize seedlings expressed five mitochondrial proteins (19, 20, 22, 23, and 28 kDa), whereas only one was detected in wheat and rye, which may account for maize’s superior heat tolerance [242]. This difference occurs because maize possesses a more robust mitochondrial stress response system. The expression of multiple mitochondrial heat-responsive proteins enhances its ability to maintain energy production, protect mitochondrial structure and prevent oxidative damage under high temperatures [243]. In contrast, wheat and rye express fewer such proteins, limiting their capacity to stabilize mitochondrial function and thus reducing their overall heat tolerance [244]. In addition, HSP68, a mitochondrial precursor protein, shows increased synthesis under elevated temperatures, emphasizing mitochondrial HSPs in thermotolerance [245,246].

HSPs play a critical protective role in preserving the protein biosynthesis machinery during heat stress, preventing protein denaturation and ensuring continued translation. Small HSPs are especially important in preventing protein aggregation, whereas other HSPs, such as HSP68 and HSP101, have distinct and vital roles [247]. HSP101, a robust chaperone, facilitates renaturation of denatured proteins, with expression patterns varying across species and developmental stages. In maize, HSP101 is highly expressed in tassels, ears, embryos, and endosperm compared to roots and leaves [248]. In *A. thaliana*, the hot1 gene, encoding HSP101, has been shown to enhance heat tolerance [249]. Numerous studies have demonstrated the diverse roles of HSPs in plant heat tolerance. For instance, a 22 kDa HSP in *Chenopodium album* and common bean associates with chloroplast membranes, altering membrane composition, reducing fluidity, and improving ATP transport efficiency [250,251]. In pumpkin, mitochondrial HSPs have been isolated and shown to contribute significantly to heat stress responses [252]. Certain HSPs exhibit temperature-dependent subcellular localization as they accumulate in the cytosol at 27 °C and in chloroplasts at 43 °C, implicating them in protecting photosynthetic machinery [253]. Similarly, the elongation factor Tu (EF-Tu, 45–46 kDa) protects the chloroplast stroma in maize under heat stress [254].

Rapid HSP accumulation is crucial for maintaining the integrity of cellular metabolic machinery. Plants adapted to semi-arid and arid environments exhibit accelerated HSP synthesis to cope with high leaf temperatures [53]. In soybean seedlings, HSPs regulate the conformational state of client proteins, modulating their function under stress [255]. Variant HSP forms, such as HSP64 kDa and HSP72 kDa, are also induced under heat stress [256]. Overexpression of HSP70, for instance, enhances heat tolerance in young pea seedlings [257].

#### 2.5.2. Role of Dehydrins in Heat Stress Response

Dehydrins (DHNs) constitute a major subfamily of the late embryogenesis abundant (LEA) D-11 protein family [258]. LEA proteins were first identified in cotton cotyledons, where stage-specific changes in mRNA and protein abundance were observed across 18 distinct LEA families [259]. DHNs are typically synthesized during late seed development and in response to dehydration-related stresses, including heat stress [260]. In addition, cloned DHN genes include the cotton D-11 gene [261], rice RAB16 (ABA-responsive), and maize RAB17 [262]. Notably, DHN expression is not restricted to higher plants but has also been reported in cyanobacteria, brown algae, ferns, and conifers [263,264]. A recent study investigated the function of the *DHN4* gene in barley under various stresses. Transcription of *DHN4* was significantly upregulated in the landrace Rihane in response to dehydration stress, compared to the landrace Manel [265]. Additionally, recombinant RhDHN4 protein was heat-stable but susceptible to protease digestion, characteristic of intrinsically disordered proteins [266]. Functionally, RhDHN4 protected lactate dehydrogenase (LDH) from heat-induced denaturation and prevented aggregation of the leaf proteome [267]. Overexpression of RhDHN4 in yeast enhanced stress tolerance, mediated by the protein’s ability to self-dimerize, as confirmed by yeast two-hybrid and GST pull-down assays. These findings demonstrate that RhDHN4 plays a key role in enhancing heat stress tolerance in barley [265]. Subcellular localization studies show that DHNs are distributed across the nucleus, cytoplasm, mitochondria and chloroplasts [263]. Under heat stress, DHNs associate with cytoplasmic membranes, helping to maintain their stability and integrity. By preventing lipid peroxidation and protein denaturation, DHNs protect cellular structures and functions, thereby enhancing plant thermotolerance and overall resilience to stress [268]. In maize, mature embryos accumulate substantial DHN levels during development [269], and in sugarcane, three low molecular weight DHNs are induced in leaves, contributing to heat tolerance [268]. Plant responses to dehydrative stress, including heat, span all developmental stages, potentially affecting flowering time, tillering and overall growth.

#### 2.5.3. Transcriptomics and Proteomics Analyses

Extensive research has examined transcriptomic responses of plants under combined stress conditions, including heat stress, revealing conserved patterns of gene regulation across species [270]. These studies highlight key genes and gene families critical for abiotic stress tolerance, providing valuable insights for developing heat-resilient crops [271]. For instance, Liu et al. (2020) identified *OsNTL3*, which encodes a processed protein form inducibly expressed during the seedling-stage in rice [272]. Its expression is constitutively regulated by endoplasmic reticulum stress, enhancing thermotolerance. Similarly, transcriptomic analyses in soybean under heat stress revealed upregulation of genes involved in oxidation-reduction processes, protein folding and small molecule metabolism [273].

**Figure 2:**
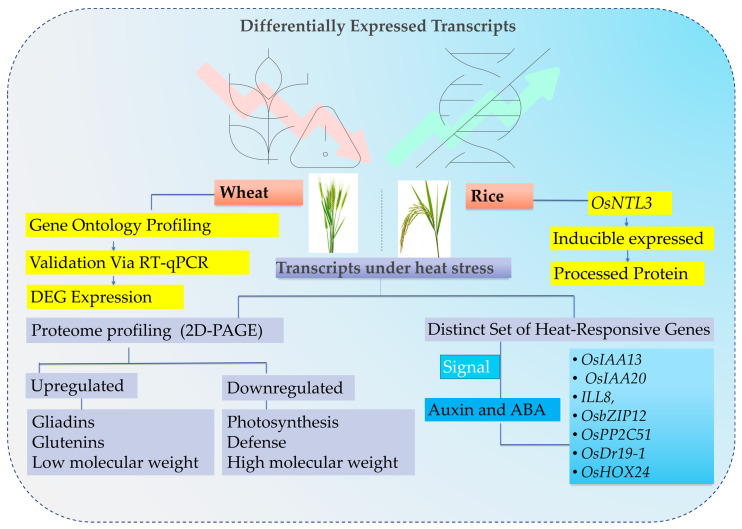
Schematic representation illustrating transcriptomic changes and gene expression patterns in plants in response to environmental stress conditions.

In wheat, the effects of daytime only and combined day–night heat stress during the grain-filling stage have been examined using gene expression analysis and proteomic profiling [274]. Gene expression was evaluated by real-time quantitative PCR (RT-qPCR), focusing on genes involved in starch biosynthesis, starch transport, transcriptional regulation, stress responses and nutrient storage [275]. Moreover, analyses across four stages of grain development revealed the activation of multiple physiological pathways (Figure 2). Under optimal conditions, gene expression continued up to 28 days after anthesis (DAA) [276].

Proteomic profiling using two-dimensional polyacrylamide gel electrophoresis (2D-PAGE) and matrix-assisted laser desorption/ionization time-of-flight mass spectrometry (MALDI-TOF MS/MS) revealed significant changes in protein abundance under heat stress [277]. Proteins associated with translation, gliadins and low-molecular-weight (LMW) glutenins were upregulated, while those involved in glycolysis, photosynthesis, defense, and high-molecular-weight (HMW) glutenins were downregulated [278]. Moreover, overall, daytime heat stress accelerated defense responses by advancing gene expression, whereas combined day-and-night stress induced broader suppression across multiple regulatory pathways [279]. Daytime heat stress primarily accelerates plant defense mechanisms by triggering the early activation of stress-responsive genes. This rapid induction helps plants cope with elevated temperatures during the day, allowing protective proteins and metabolites to accumulate when stress is most intense [280]. In contrast, combined day-and-night heat stress imposes a more severe and continuous challenge. Under these conditions, plants often experience energy depletion and metabolic strain, suppressing multiple regulatory pathways, including those involved in growth, signaling and stress response [281]. As a result, instead of selectively activating defense genes, the plant broadly downregulates gene expression, reflecting a systemic stress response. These molecular and physiological changes contributed to a shortened grain-filling period, reduced grain weight, lower yield, and impaired processing quality [282].

HSFs are pivotal in enhancing plant thermotolerance. Transcriptome analyses in wheat genotypes AS3809, PDW274 and PBW725 revealed approximately 74,000, 68,000, and 76,000 expressed genes, respectively [283]. In addition, gene Ontology (GO) profiling demonstrated strong conservation of biological, molecular and cellular functions across all three genotypes, indicating functional stability within wheat transcriptomes [284]. These results suggest that many differentially expressed genes (DEGs) contribute to heat stress tolerance. Moreover, validation via RT-qPCR confirmed the consistency of DEG expression patterns with sequencing data, underscoring the reliability of the transcriptome analysis [285]. Collectively, these findings (Figure 2) provide key insights into the molecular mechanisms underlying thermotolerance in wheat [286].

In rice, transcriptome analysis of the red rice cultivar Annapurna identified a distinct set of heat-responsive genes and pathways, particularly associated with auxin and ABA signaling [271]. RT-qPCR validation confirmed the expression of auxin and ABA-related genes, including *OsIAA13*, *OsIAA20*, *ILL8*, *OsbZIP12*, *OsPP2C51*, *OsDi19*-1, and *OsHOX24*, under high-temperature conditions [287,288]. Additionally, auxin inducible SAUR genes were also significantly upregulated at elevated temperatures. By comparing genes with opposing expression patterns under heat stress, a regulatory network was constructed involving transcription factors (TFs) such as HSFs, NAC, WRKY, bHLH, and bZIPs, alongside their corresponding target genes [289]. This network provides insights into the coordinated regulation of temperature-responsive genes in rice, offering a valuable resource for identifying candidate genes linked to thermotolerance and temperature sensing mechanisms [290,291]. In another study, the heat-sensitive Indian wheat cultivar PBW343 was subjected to heat stress at 42 °C for 2 h, and transcriptome analysis via RNA sequencing (RNA-seq) identified 160 differentially expressed transcripts, 143 upregulated and 17 downregulated [292]. The result reflects a predominant activation of protective and compensatory mechanisms. The lower number of downregulated genes suggests that most cellular machinery remains active or is enhanced to mitigate heat-induced damage rather than being suppressed. Among these, Rca1β was selected for further functional investigation and overexpression studies to explore its role in heat stress adaptation [293].

#### 2.5.4. Epigenetic Modifications Regulate Plant Responses and Adaptation During Heat Stress

Recent studies have highlighted the critical role of epigenetic modifications in enabling crop plants to respond to heat stress. These modifications regulate rapid gene expression changes and longer-term ‘stress memory’ enhancing plant tolerance during recurrent heat episodes [291]. Elevated temperatures often induce rapid changes in DNA methylation, with widespread demethylation in the promoters of heat-responsive genes, enabling swift activation of protective pathways [294]. Concurrently, heat stress induces post-translational modifications of histone proteins. Additionally, active chromatin marks, such as H3K4 trimethylation and H3 acetylation, accumulate at heat shock protein loci, whereas repressive marks like H3K27 trimethylation are removed [295,296]. These changes relax the nucleosome structure, enabling easier access to DNA by the transcriptional machinery [297]. This happens because plants need to respond quickly to heat stress. Under normal conditions, heat shock protein genes are kept inactive by repressive chromatin marks like H3K27 trimethylation. When temperatures rise, these repressive marks are removed while active marks such as H3K4 trimethylation and H3 acetylation accumulate, loosening the chromatin and making the DNA accessible to transcription machinery. This dynamic shift allows rapid activation of heat shock protein genes, enabling the plant to produce protective proteins immediately and cope with heat-induced cellular damage [297].

Chromatin-remodeling complexes further enhance this response by repositioning or removing nucleosomes at key stress-responsive promoters, thereby facilitating defense gene activation [298]. Noncoding RNAs, particularly stress-induced microRNAs and long noncoding RNAs, also contribute by directing histone-modifying enzymes or targeting specific transcripts for degradation [299,300]. Some of these epigenetic modifications persist after the stress has subsided, creating a primed chromatin state that allows a faster and stronger response to subsequent heat stress [301]. Remarkably, such epigenetic ‘stress memory’ can sometimes be transmitted across generations, providing offspring with a transgenerational advantage under heat stress conditions [302].

**Figure 3:**
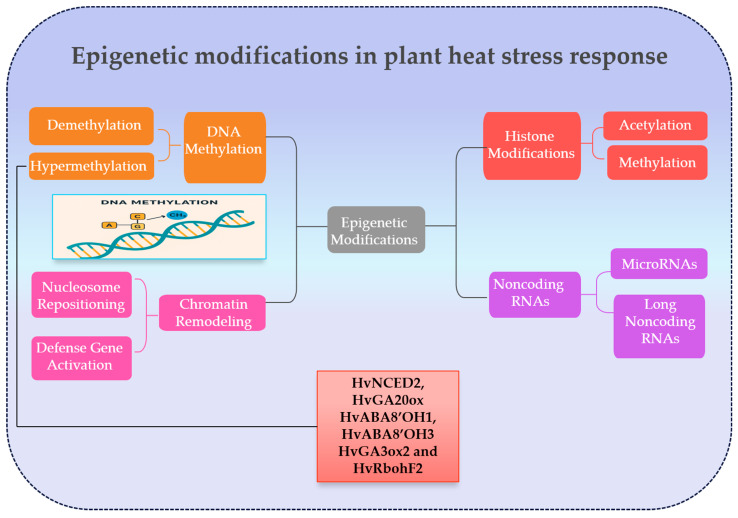
Schematic representation illustrating epigenetic modifications and regulatory mechanisms in plants responding to heat stress conditions for improved tolerance.

Moreover, the interconnected layers of epigenetic regulation comprising DNA methylation, histone modifications, chromatin remodeling, and noncoding RNAs form a dynamic and heritable regulatory network that enables crop plants to perceive, memorize, and adapt to elevated temperature conditions [303]. This highlights the potential of epigenetic manipulation as a strategy for developing heat-tolerant crop varieties [301]. RNA-directed DNA methylation (RdDM) represents a distinctive plant-specific epigenetic pathway, wherein noncoding RNAs guide DNA methylation to enhance heat stress tolerance. Disruption of RdDM components increases plant susceptibility to heat stress (Figure 3) [304,305]. Histone modifications also play critical roles in acquired thermotolerance. For instance, the histone chaperone ASF1 supports thermotolerance by facilitating H3K56 deacetylation, while the FGT1–BRM/CHR11/CHR17 complex regulates nucleosome positioning, thereby reinforcing stress-responsive chromatin architecture [301]. HSFA2 is pivotal in maintaining sustained expression of heat shock protein genes by recruiting histone methyltransferases to memory loci, thereby establishing a transcriptional “heat memory” during repeated stress events [10].

In seeds, GA and ROS generated by NADPH oxidases stimulate germination, whereas ABA acts as an inhibitory signal [306]. Under heat stress, the expression of ABA biosynthetic genes such as *HvNCED1* and *HvNCED2* is markedly downregulated, while genes associated with ABA catabolism (*HvABA8′OH1*), GA biosynthesis such as HvGA20ox, HvGA3ox and ROS production (HvRbohF2) are significantly upregulated during seed imbibition [307]. Methylated DNA immunoprecipitation followed by qPCR (MeDIP-qPCR) revealed epigenetic alterations at key regulatory loci: promoters of *HvNCED* genes were hypermethylated, whereas promoters of *HvABA8′OH1*, *HvABA8′OH3*, *HvGA3ox2*, and *HvRbohF2* were hypomethylated in heat-treated seeds [274]. These modifications suggest that heat stress during grain filling induces locus-specific DNA methylation changes, enhancing seed germination potential in barley [308].

Similarly, in upland cotton (*Gossypium hirsutum*) both short- and long-term heat stress significantly inhibited seedling growth and triggered dynamic epigenetic changes [309]. Heat exposure altered histone H3K4 dimethylation (H3K4me2) and H4K5 acetylation (H4K5ac) patterns. Chromatin immunoprecipitation-qPCR (ChIP-qPCR) revealed a rapid increase in H3K4me2 during short-term heat stress, whereas H4K5ac levels gradually increased across both short- and long-term treatments [309]. These histone modifications were closely associated with the expression of key heat-responsive genes, including GhHSFA1a, GhHSFA2, GhHSP3, GhRBCS, GhERF1A, and GhHXK1 [310]. Heat stress also induced DNA methylation changes at the GhHSFA1a promoter, correlating with transcriptional activation. Collectively, these findings indicate that the coordinated regulation of H3K4me2, H4K5ac and DNA methylation fine-tune the expression of critical heat-responsive genes contributing to thermo tolerance in cotton [301].

Histone modifications are key regulators of gene expression during heat stress. Enzymes such as histone acetyltransferases (HATs), methyltransferases, deacetylases, and demethylases dynamically modify chromatin structure and transcriptional activity [295]. For example, the HAT GCN5 enhances thermotolerance by increasing H3K9/K14 acetylation at the promoters of HSFA3 and UVH6 [311]. Conversely, histone deacetylases (HDACs) like HD2C repress thermotolerance by deacetylating H4K16 and interacting with SWI3B, a subunit of the SWI/SNF chromatin-remodeling complex, thereby suppressing key heat shock response genes such as HSFA3 and HSP101 [312]. Similarly, HDA6 contributes to thermotolerance through RdDM-mediated gene suppression [313]. Heat stress also triggers subcellular relocalization of regulatory proteins: the nucleoporin HOS1 and the phosphatase regulated HDAC, HDA9 (controlled by PP2AB0b) translocate from the cytoplasm to the nucleus during heat exposure, where they participate in amplifying and modulating the heat shock signaling cascade [314,315].

## 3. Conclusions

Heat stress is a major environmental constraint that severely affects crop growth, development and yield. Its impact varies with climatic zone, duration and timing of exposure, all of which influence the extent of yield reduction across crop species. Global warming and anthropogenic activities are primary drivers of rising temperatures that trigger heat stress. Nevertheless, plants possess intrinsic adaptive mechanisms, adjusting cellular and molecular processes to survive elevated temperatures. Although plant responses to heat stress have been widely investigated across developmental stages, the complex molecular and physiological mechanisms remain partially understood. Ongoing climate change, marked by seasonal temperature shifts and greater diurnal fluctuations, further complicates assessing and managing heat stress effects.

Genetic variation in heat tolerance exists within and among plant species, providing breeding and genetic improvement opportunities. Plants employ diverse metabolic pathways and physiological processes to enhance survival under high temperature stress. Early studies on heat tolerance primarily examined structural, morphological, physiological, and molecular responses to uncover underlying mechanisms. Physiologically, plants activate cooling strategies when exposed to high temperatures. Stomatal regulation of transpiration promotes leaf cooling and stomatal opening under elevated ambient temperatures, helping lower leaf temperature. At the molecular level, HSPs are central to thermotolerance. These molecular chaperones are rapidly produced in response to heat, stabilizing and refolding denatured proteins, preventing aggregation, and aiding protein degradation. Key HSP families such as HSP70, HSP90, and sHSPs are critical for maintaining cellular homeostasis during thermal stress. Their expression is chiefly controlled by HSFs, which are activated by heat sensing and bind to HSEs in HSP gene promoters.

In addition, epigenetic modifications such as DNA methylation, histone modifications, and noncoding RNAs are critical regulators of gene expression under heat stress. These changes can remodel chromatin structure and accessibility, thereby modulating the transcription of stress-responsive genes. Epigenetic memory may also confer transgenerational tolerance, enabling progeny to exhibit enhanced resilience to ancestral stress. Understanding gene expression patterns and their functional roles during plant development is vital for designing targeted strategies to improve heat-stress tolerance. At the field level, optimizing sowing time and method, scheduling irrigation, and cultivating heat-tolerant genotypes can substantially reduce yield losses. A holistic approach that combines molecular, biochemical, and morphological insights with optimized agronomy is essential for strengthening crop resilience to heat stress.

Significant knowledge gaps persist despite substantial advances in understanding plant heat stress responses. One of the most critical challenges is the trade-off between growth and thermotolerance. Enhanced stress tolerance often depends on energy-demanding protective mechanisms such as synthesizing HSPs, osmolytes and antioxidants, which can divert essential resources away from growth and yield formation. The molecular and physiological mechanisms regulating this balance remain poorly understood. Furthermore, there is limited insight into how plants dynamically reallocate energy and assimilate under fluctuating thermal regimes and how these adjustments influence long-term adaptation, fitness and productivity. Addressing these gaps through integrative modeling and field-based phenotyping could help elucidate the underlying trade-offs and ultimately decouple thermotolerance from yield penalties, facilitating the development of heat-resilient yet high-yielding cultivars.

Although molecular breeding has greatly advanced the identification and introgression of heat tolerance traits, several constraints limit its broader application. Current approaches often rely on QTLs or candidate genes identified under controlled environments, which may not consistently perform under variable field conditions due to strong genotype × environment interactions. Moreover, the polygenic and complex nature of heat tolerance, encompassing multiple signaling pathways, transcriptional networks and epigenetic modifications, reduces the efficiency of marker-assisted selection focused on single loci. The narrow genetic diversity within elite breeding pools further restricts access to novel adaptive alleles. High-throughput phenotyping and functional validation also lag behind genomic discoveries, creating a persistent disconnect between molecular markers and field-level performance. While integrating multi-omics datasets with precision phenotyping, genomic prediction and genome editing offers great promise, the effective translation of molecular insights into durable, field-relevant thermotolerance remains a major challenge for modern breeding programs.

## Figures and Tables

**Table 1:** Impacts of heat stress on plant reproductive phase with effective temperature for survival to plants.

Crop	Growth Stage	Control Temperature	Extreme Temperature	Plant Response Studied	References
		(Degree Celsius)	(Degree Celsius)		
Rice (*Oryza Sativa*)	Inflorescence development	25	37	Pollen sterility	[152]
	Microsporogenesis	28	33	Reduced pollen production, Pollen inviability	[152]
	Pollen maturation	28	39	Down regulation of expression of tapetum genes	[153]
	Anthesis	30	>33.7	Pollen sterility	[154]
	Pollination	28	38	Spikelet sterility	[155]
Wheat (*T. aestivum*)	Inflorescence initiation	25	30	Early anthesis	[156]
	Inflorescence development	26	33	Meiotic abnormalities	[157]
	Microsporogenesis	20	30	Male sterility	[158]
	Anthesis	28	38	Less grains per ear	[159]
	Post anthesis	18	30	Reduced kernel weight	[160]
	Post fertilization	20	35	Yield reduction	[161]
Barley (*Hordeum vulgare*)	Inflorescence development	20	30	Pollen inviability	[162]
	Microsporogenesis	20	30	Abnormal microspores	[163]
	Microgametogenesis	20	30	Pollen abortion	[164]
	Pollen maturation	20	30	Anther wall degradation	[165]
	Post anthesis	20	40	Reduced grain weight	[166]
Brachypodium (*Brachypodiumd istachyon*)	Inflorescence initiation	24	32	Less tillering	[167]
	Microgametogenesis	24	36	Pollen development ceases	[168]
	Anthesis	24	36	Anther indehiscence	[168]
	Pre-fertilization	24	32	Reduce pollen germination	[168]
	Pollination	22	27	Reduced grain weight	[168]
MAIZE (*Zea mays*)	Inflorescence development	33.9	>35	Male and female sterility	[104]
	Anthesis	27	38	Reduced pollen germination	[169]
	Pollination	27	38	Poor kernel set	[169]
	Pre silking	25	35	Decrease in ear weight	[170]
Sorghum (*Sorghum bicolor*)	Inflorescence initiation	25	37	Floret sterility	[171]
	Inflorescence development	30	38	Reduced pollen germination	[172]
	Anthesis	28	33	Embryo abortion	[173]
	Post anthesis	32	40	Lesser grain yield	[174]
Pearl millet (*Pennisetumglacum*)	Inflorescence development	35	>42	Reduced seed set	[175]
Arabidopsis (*A. thaliana*)	Inflorescence development	22	42	Pollen release is impaired	[176]
Peas (*Pisum sativum*)	Inflorescence development	20	33	Abortion of floral buds	[177]
	Inflorescence development	20	30	Less flowering nodes	[177]
	Anthesis	20	28	Lesser number of seeds per pod	[177]
	Post fertilization	24	32	Reduction in yield	[140]
Tomato (*Solanum lycopersicum*)	Inflorescence initiation	28	>29	Reduction in fruit yield	[178]
	Inflorescence development	18	28	Reduce Stigma Surface Area	[178]
	Microsporogenesis	28	32	Reduced expression of Proline Transporter I	[179]
	Microgametogenesis	28	35	Stigma exertion without anthesis	[180]
	Pollen maturation	25	29	Decrease fruit set	[181]
	Anthesis	28	32	Inviable pollen	[182]

## Data Availability

No new data were created or analyzed in this study. Data sharing is not applicable to this article.

## References

[1] Jagadish S.V.K., Way D.A., Sharkey T.D. (2021). Plant Heat Stress: Concepts Directing Future Research. Plant Cell Environ..

[2] Ugah E.P.T.A., Ndubuisi O.G., FNisafetyE F.I.S.P.O.N. (2025). The Effects of Greenhouse Gas Emissions on Global Climate Patterns. Int. J. Innov. Biochem. Microbiol. Res..

[3] Zhao C., Liu B., Piao S., Wang X., Lobell D.B., Huang Y., Huang M., Yao Y., Bassu S., Ciais P. (2017). Temperature increase reduces global yields of major crops in four independent estimates. Proc. Natl. Acad. Sci. USA.

[4] Zhao W., Chou J., Li J., Xu Y., Li Y., Hao Y. (2022). Impacts of Extreme Climate Events on Future Rice Yields in Global Major Rice-Producing Regions. Int. J. Environ. Res. Public Health.

[5] Chhabra S., Maheshwari C., Umar S., Khan M.I.R. (2025). Mitigating High-Temperature Stress in Plants: Strategies for Sustaining Crop Growth and Yield. Optimizing Plant Health Under Abiotic-Stress Environments.

[6] Sachdev S., Ansari S.A., Ansari M.I., Fujita M., Hasanuzzaman M. (2021). Abiotic Stress and Reactive Oxygen Species: Generation, Signaling, and Defense Mechanisms. Antioxidants.

[7] Sehgal A., Sita K., Siddique K.H.M., Kumar R., Bhogireddy S., Varshney R.K., HanumanthaRao B., Nair R.M., Prasad P.V.V., Nayyar H. (2018). Drought or/and Heat-Stress Effects on Seed Filling in Food Crops: Impacts on Functional Biochemistry, Seed Yields, and Nutritional Quality. Front. Plant Sci..

[8] Nadeem M., Li J., Wang M., Shah L., Lu S., Wang X., Ma C. (2018). Unraveling Field Crops Sensitivity to Heat Stress: Mechanisms, Approaches, and Future Prospects. Agronomy.

[9] Charney D.S. (2004). Psychobiological Mechanisms of Resilience and Vulnerability: Implications for Successful Adaptation to Extreme Stress. AJP.

[10] Nishio H., Kawakatsu T., Yamaguchi N. (2024). Beyond Heat Waves: Unlocking Epigenetic Heat Stress Memory in *Arabidopsis*. Plant Physiol..

[11] Barnabás B., Jäger K., Fehér A. (2008). The Effect of Drought and Heat Stress on Reproductive Processes in Cereals. Plant Cell Environ..

[12] Lenaz G., Castelli G.P. (1985). Membrane Fluidity: Molecular Basis and Physiological Significance. Structure and Properties of Cell Membrane Structure and Properties of Cell Membranes.

[13] Devireddy A.R., Tschaplinski T.J., Tuskan G.A., Muchero W., Chen J.G. (2021). Role of Reactive Oxygen Species and Hormones in Plant Responses to Temperature Changes. Int. J. Mol. Sci..

[14] Singh A., Mehta S., Yadav S., Nagar G., Ghosh R., Roy A., Chakraborty A., Singh I.K. (2022). How to Cope with the Challenges of Environmental Stresses in the Era of Global Climate Change: An Update on ROS Stave off in Plants. Int. J. Mol. Sci..

[15] Bokszczanin K.L., Fragkostefanakis S., Solanaceae Pollen Thermotolerance Initial Training Network (SPOT-ITN) Consortium (2013). Perspectives on Deciphering Mechanisms Underlying Plant Heat Stress Response and Thermotolerance. Front. Plant Sci..

[16] Kang X., Zhao L., Liu X. (2024). Calcium Signaling and the Response to Heat Shock in Crop Plants. Int. J. Mol. Sci..

[17] Szaker H.M., Gyula P., Szittya G., Csorba T. (2020). Regulation of High-Temperature Stress Response by Small RNAs. Plant microRNAs: Shaping Development and Environmental Responses.

[18] Bhardwaj R., Lone J.K., Pandey R., Mondal N., Dhandapani R., Meena S.K., Khan S., Gayacharan (2023). Insights into Morphological and Physio-Biochemical Adaptive Responses in Mungbean (*Vigna radiata* L.) under Heat Stress. Front. Genet..

[19] Hasanuzzaman M., Nahar K., Alam M.M., Roychowdhury R., Fujita M. (2013). Physiological, Biochemical, and Molecular Mechanisms of Heat Stress Tolerance in Plants. Int. J. Mol. Sci..

[20] Hussain S., Ulhassan Z., Brestic M., Zivcak M., Zhou W., Allakhverdiev S.I., Yang X., Safdar M.E., Yang W., Liu W. (2021). Photosynthesis Research under Climate Change. Photosynth. Res..

[21] Kathpalia R., Bhatla S.C., Bhatla S.C., A. Lal M. (2018). Plant Water Relations. Plant Physiology, Development and Metabolism.

[22] Buckley T.N. (2019). How Do Stomata Respond to Water Status?. New Phytol..

[23] Chachar S., Ahmed N., Hu X. (2025). Drought-Induced Aesthetic Decline and Ecological Impacts on Ornamentals: Mechanisms of Damage and Innovative Strategies for Mitigation. Plant Biol..

[24] Prasad P.V.V., Djanaguiraman M., Stewart Z.P., Ciampitti I.A. (2020). Agroclimatology of Maize, Sorghum, and Pearl Millet. Agroclimatology: Linking Agriculture to Climate.

[25] Sandhu N., Aggarwal H., Kumar A., Augustine G., Vishnoi R., Pandey A.K., Chauhan H., Chhuneja P. (2025). Regulating Plant Architecture to Enhance the Future of Cereal Crop Production. Physiol. Plant..

[26] Gruda N.S., Samuolienė G., Dong J., Li X. (2025). Environmental Conditions and Nutritional Quality of Vegetables in Protected Cultivation. Compr. Rev. Food Sci. Food Saf..

[27] Lakshmanan P., Robinson N. (2013). Stress Physiology: Abiotic Stresses. Sugarcane: Physiology, Biochemistry, and Functional Biology.

[28] Fan C., Hou M., Si P., Sun H., Zhang K., Bai Z., Wang G., Li C., Liu L., Zhang Y. (2022). Response of Root and Root Hair Phenotypes of Cotton Seedlings under High Temperature Revealed with RhizoPot. Front. Plant Sci..

[29] Vaughn L.M., Baldwin K.L., Jia G., Verdonk J.C., Strohm A.K., Masson P.H., Liu B. (2011). The Cytoskeleton and Root Growth Behavior. The Plant Cytoskeleton.

[30] Blume Y.B., Krasylenko Y.A., Yemets A.I. (2017). The Role of the Plant Cytoskeleton in Phytohormone Signaling under Abiotic and Biotic Stresses. Mechanism of Plant Hormone Signaling Under Stress.

[31] Calleja-Cabrera J., Boter M., Oñate-Sánchez L., Pernas M. (2020). Root Growth Adaptation to Climate Change in Crops. Front. Plant Sci..

[32] Waqas M.A., Wang X., Zafar S.A., Noor M.A., Hussain H.A., Nawaz M.A., Farooq M. (2021). Thermal Stresses in Maize: Effects and Management Strategies. Plants.

[33] Arachchige S.M., Razzaq A., Dai H.Y., Wang J. (2024). Confronting Heat Stress in Crops Amid Global Warming: Impacts, Defense Mechanisms, and Strategies for Enhancing Thermotolerance. Crop Breed. Genet. Genom..

[34] Khanzada A., Yan K., Hu W., Malko M., Khan K.A., Bao Y., Elboughdiri N., Li Y. (2025). Heat Stress Response Mechanisms and Resilience Strategies in Wheat. J. Agron. Crop Sci..

[35] Djanaguiraman M., Narayanan S., Erdayani E., Prasad P.V.V. (2020). Effects of High Temperature Stress during Anthesis and Grain Filling Periods on Photosynthesis, Lipids and Grain Yield in Wheat. BMC Plant Biol..

[36] Beddington J.R., Asaduzzaman M., Fernández A., Clark M.E., Guillou M., Jahn M.M., Erda L., Mamo T., Bo N.V., Nobre C.A. (2012). Achieving Food Security in the Face of Climate Change: Final Report from the Commission on Sustainable Agriculture and Climate Change.

[37] Grossiord C., Buckley T.N., Cernusak L.A., Novick K.A., Poulter B., Siegwolf R.T.W., Sperry J.S., McDowell N.G. (2020). Plant Responses to Rising Vapor Pressure Deficit. New Phytol..

[38] Marquez D., Gardner A., Busch F. (2025). Navigating Challenges in Interpreting Plant Physiology Responses through Gas Exchange Results in Stressed Plants. Plant Ecophysiol..

[39] Lin H., Chen Y., Zhang H., Fu P., Fan Z. (2017). Stronger Cooling Effects of Transpiration and Leaf Physical Traits of Plants from a Hot Dry Habitat than from a Hot Wet Habitat. Funct. Ecol..

[40] Zhao C., Haigh A.M., Holford P., Chen Z.H. (2018). Roles of Chloroplast Retrograde Signals and Ion Transport in Plant Drought Tolerance. Int. J. Mol. Sci..

[41] Batool I., Ayyaz A., Qin T., Wu X., Chen W., Hannan F., Zafar Z.U., Naeem M.S., Farooq M.A., Zhou W. (2025). Morphological, Physiological, and Molecular Responses to Heat Stress in Brassicaceae. Plants.

[42] Hasanuzzaman M., Nahar K., Gill S.S., Fujita M. (2013). Drought Stress Responses in Plants, Oxidative Stress, and Antioxidant Defense. Climate Change and Plant Abiotic Stress Tolerance.

[43] Khan S., Saify S., Sofo A., Khan N.A. (2024). The Mechanisms of Melatonin Action in Shielding Photosynthesis during Heat Stress. CABI Rev..

[44] Sahoo R., Samanta D., Sow S., Ranjan S., Nath D., Sadhu S., Kumar N., Rana L., Kumar A., Roy D.K. (2025). Nexus between Photosynthesis and Radiation Use Efficiency towards Achieving Sustainability in the Era of Climate Change: An Overview. Discov. Environ..

[45] Venios X., Korkas E., Nisiotou A., Banilas G. (2020). Grapevine Responses to Heat Stress and Global Warming. Plants.

[46] Chen J.H., Tang M., Jin X.Q., Li H., Chen L.S., Wang Q.L., Sun A.Z., Yi Y., Guo F.Q. (2022). Regulation of Calvin–Benson Cycle Enzymes under High Temperature Stress. Abiotech.

[47] Qiao M., Hong C., Jiao Y., Hou S., Gao H. (2024). Impacts of Drought on Photosynthesis in Major Food Crops and the Related Mechanisms of Plant Responses to Drought. Plants.

[48] Ranawana S.R.W.M.C.J.K., Bramley H., Palta J.A., Siddique K.H.M., Harohalli Masthigowda M., Gopalareddy K., Khobra R., Singh G., Pratap Singh G. (2023). Role of Transpiration in Regulating Leaf Temperature and Its Application in Physiological Breeding. Translating Physiological Tools to Augment Crop Breeding.

[49] Sullivan C.Y., Norcio N.V., Eastin J.D., Muhammed A., Aksel R., von Borstel R.C. (1977). Plant Responses to High Temperatures. Genetic Diversity in Plants.

[50] Rogowska A., Szakiel A. (2020). The Role of Sterols in Plant Response to Abiotic Stress. Phytochem. Rev..

[51] Li X., Zhu L., Wang H., Zhou X., Wang M., Li L., Liu F., Sun J., Xiao G. (2025). Peptide Hormone-Mediated Regulation of Plant Development and Environmental Adaptability. Adv. Sci..

[52] Lee Z., Lim J.A., Harikrishna J.A., Islam T., Abd Rahim M.H., Yaacob J.S. (2024). Regulation of Plant Responses to Temperature Stress: A Key Factor in Food Security and for Mitigating Effects of Climate Change. Int. J. Plant Prod..

[53] Ahmad M., Waraich E.A., Skalicky M., Hussain S., Zulfiqar U., Anjum M.Z., Habib ur Rahman M., Brestic M., Ratnasekera D., Lamilla-Tamayo L. (2021). Adaptation Strategies to Improve the Resistance of Oilseed Crops to Heat Stress Under a Changing Climate: An Overview. Front. Plant Sci..

[54] Zheng Y., Cai Z., Wang Z., Maruza T.M., Zhang G. (2025). The Genetics and Breeding of Heat Stress Tolerance in Wheat: Advances and Prospects. Plants.

[55] Banerjee A., Roychoudhury A. (2017). Abiotic Stress, Generation of Reactive Oxygen Species, and Their Consequences. Reactive Oxygen Species in Plants.

[56] Dar M.I., Naikoo M.I., Khan F.A., Rehman F., Green I.D., Naushin F., Ansari A.A., Khan M.I.R., Khan N.A. (2017). An Introduction to Reactive Oxygen Species Metabolism Under Changing Climate in Plants. Reactive Oxygen Species and Antioxidant Systems in Plants: Role and Regulation Under Abiotic Stress.

[57] Rawat N., Singla-Pareek S.L., Pareek A. (2021). Membrane Dynamics during Individual and Combined Abiotic Stresses in Plants and Tools to Study the Same. Physiol. Plant..

[58] Sivakumar T., Kumar A.K. (2024). An Updated Review On-Heat Stress Imbalance and Enhancement of Wheat. J. Xidian Univ..

[59] Sahoo R., Samanta D., Sow S., Ranjan S., Nath D., Sadhu S., Kumar N., Rana L., Kumar A., Roy D.K. (2022). Lipid Metabolism in Plants under Low-Temperature Stress: A Review. Physiological Processes in Plants Under Low Temperature Stress.

[60] Dhaliwal L.K., Angeles-Shim R.B. (2022). Cell Membrane Features as Potential Breeding Targets to Improve Cold Germination Ability of Seeds. Plants.

[61] Malik J.A., Lone R. (2021). Heat shock proteins with an emphasis on HSP 60. Mol. Biol. Rep..

[62] Fujita M., Hasanuzzaman M. (2022). Approaches to Enhancing Antioxidant Defense in Plants. Antioxidants.

[63] Pattnaik D., Dash D., Mishra A., Padhiary A.K., Dey P., Dash G.K. (2021). Emerging Roles of Osmoprotectants in Alleviating Abiotic Stress Response Under Changing Climatic Conditions. Climate Impacts on Sustainable Natural Resource Management.

[64] Kumar H., Chugh V., Kumar M., Gupta V., Prasad S., Kumar S., Singh C.M., Kumar R., Singh B.K., Panwar G. (2023). Investigating the Impact of Terminal Heat Stress on Contrasting Wheat Cultivars: A Comprehensive Analysis of Phenological, Physiological, and Biochemical Traits. Front. Plant Sci..

[65] Chaudhary S., Devi P., Bhardwaj A., Jha U.C., Sharma K.D., Prasad P.V., Siddique K.H., Bindumadhava H., Kumar S., Nayyar H. (2020). Identification and Characterization of Contrasting Genotypes/Cultivars for Developing Heat Tolerance in Agricultural Crops: Current Status and Prospects. Front. Plant Sci..

[66] Senguttuvel P., Jaldhani V., Raju N.S., Balakrishnan D., Beulah P., Bhadana V.P., Mangrauthia S.K., Neeraja C.N., Subrahmanyam D., Rao P.R. (2022). Breeding Rice for Heat Tolerance and Climate Change Scenario; Possibilities and Way Forward. A Review. Arch. Agron. Soil Sci..

[67] Ul Hassan M., Rasool T., Iqbal C., Arshad A., Abrar M., Abrar M.M., Habib-ur-Rahman M., Noor M.A., Sher A., Fahad S. (2022). Linking Plants Functioning to Adaptive Responses Under Heat Stress Conditions: A Mechanistic Review. J. Plant Growth Regul..

[68] Sadok W., Lopez J.R., Smith K.P. (2021). Transpiration Increases under High-Temperature Stress: Potential Mechanisms, Trade-Offs and Prospects for Crop Resilience in a Warming World. Plant Cell Environ..

[69] Hafeez U., Ali M., Hassan S.M., Akram M.A., Zafar A. (2023). Advances in Breeding and Engineering Climate-Resilient Crops: A Comprehensive Review. Int. J. Res. Adv. Agric. Sci..

[70] Jianing G., Yuhong G., Yijun G., Rasheed A., Qian Z., Zhiming X., Mahmood A., Shuheng Z., Zhuo Z., Zhuo Z. (2022). Improvement of Heat Stress Tolerance in Soybean (*Glycine max* L.), by Using Conventional and Molecular Tools. Front. Plant Sci..

[71] Zhang S., Ye H., Kong L., Li X., Chen Y., Wang S., Liu B. (2024). Multivariate Analysis Compares and Evaluates Heat Tolerance of Potato Germplasm. Plants.

[72] Prasad V.B.R., Govindaraj M., Djanaguiraman M., Djalovic I., Shailani A., Rawat N., Singla-Pareek S.L., Pareek A., Prasad P.V.V. (2021). Drought and High Temperature Stress in Sorghum: Physiological, Genetic, and Molecular Insights and Breeding Approaches. Int. J. Mol. Sci..

[73] Dawood M.F.A., Moursi Y.S., Amro A., Baenziger P.S., Sallam A. (2020). Investigation of Heat-Induced Changes in the Grain Yield and Grains Metabolites, with Molecular Insights on the Candidate Genes in Barley. Agronomy.

[74] Ayenan M.A.T., Danquah A., Hanson P., Ampomah-Dwamena C., Sodedji F.A.K., Asante I.K., Danquah E.Y. (2019). Accelerating Breeding for Heat Tolerance in Tomato (*Solanum lycopersicum* L.): An Integrated Approach. Agronomy.

[75] Yang Y., Yu J., Qian Q., Shang L. (2022). Enhancement of Heat and Drought Stress Tolerance in Rice by Genetic Manipulation: A Systematic Review. Rice.

[76] Dev W., Sultana F., He S., Hu D., Geng X., Du X., Iqbal B. (2025). Effects of High Temperatures on Pollen Germination and Physio-Morphological Traits in Upland Cotton (*Gossypium hirsutum* L.). J. Agron. Crop Sci..

[77] Yamori W., Hikosaka K., Way D.A. (2014). Temperature Response of Photosynthesis in C3, C4, and CAM Plants: Temperature Acclimation and Temperature Adaptation. Photosynth. Res..

[78] Singh J., Thakur J.K., Vats S. (2018). Photosynthesis and Abiotic Stress in Plants. Biotic and Abiotic Stress Tolerance in Plants.

[79] Zou M., Yuan L., Zhu S., Liu S., Ge J., Wang C. (2016). Effects of Heat Stress on Photosynthetic Characteristics and Chloroplast Ultrastructure of a Heat-Sensitive and Heat-Tolerant Cultivar of Wucai (*Brassica campestris* L.). Acta Physiol. Plant.

[80] Fahad S., Bajwa A.A., Nazir U., Anjum S.A., Farooq A., Zohaib A., Sadia S., Nasim W., Adkins S., Saud S. (2017). Crop Production under Drought and Heat Stress: Plant Responses and Management Options. Front. Plant Sci..

[81] Rath J.R., Pandey J., Yadav R.M., Zamal M.Y., Ramachandran P., Mekala N.R., Allakhverdiev S.I., Subramanyam R. (2022). Temperature-Induced Reversible Changes in Photosynthesis Efficiency and Organization of Thylakoid Membranes from Pea (*Pisum sativum*). Plant Physiol. Biochem..

[82] Zehra A., Wani K.I., Choudhary S., Naeem M., Khan M.M.A., Aftab T. (2023). Involvement of Abscisic Acid in Silicon-Mediated Enhancement of Copper Stress Tolerance in *Artemisia annua*. Plant Physiol. Biochem..

[83] Zahra S.A., Iqbal J., Abbasi B.A., Kanwal S., Alwahibi M.S., Elshikh M.S., Rizwan M., Iqbal R., Mahmood T. (2024). Phylogenetic Analysis of Selected Species of Asteraceae on the Basis of RPS 11 Gene. Sci. Rep..

[84] Yamori W. (2016). Photosynthetic Response to Fluctuating Environments and Photoprotective Strategies under Abiotic Stress. J. Plant Res..

[85] Kono M., Oguchi R., Terashima I., Lüttge U., Cánovas F.M., Risueño M.-C., Leuschner C., Pretzsch H. (2024). Photoinhibition of PSI and PSII in Nature and in the Laboratory: Ecological Approaches. Progress in Botany Vol. 84.

[86] Croce R., Carmo-Silva E., Cho Y.B., Ermakova M., Harbinson J., Lawson T., McCormick A.J., Niyogi K.K., Ort D.R., Patel-Tupper D. (2024). Perspectives on Improving Photosynthesis to Increase Crop Yield. Plant Cell.

[87] Cheng D.-D., Zhang Z.-S., Sun X.-B., Zhao M., Sun G.-Y., Chow W.S. (2016). Photoinhibition and Photoinhibition-like Damage to the Photosynthetic Apparatus in Tobacco Leaves Induced by Pseudomonas Syringae Pv. Tabaci under Light and Dark Conditions. BMC Plant Biol..

[88] Yan K., Chen P., Shao H., Shao C., Zhao S., Brestic M. (2013). Dissection of Photosynthetic Electron Transport Process in Sweet Sorghum under Heat Stress. PLoS ONE.

[89] Krause G.H. (1993). The Role of Oxygen in Photoinhibition of Photosynthesis. Causes of Photooxidative Stress and Amelioration of Defense Systems in Plants.

[90] Fahad S., Khan F.A., Pandupuspitasari N., Hussain S., Khan I.A., Saeed M., Saud S., Hassan S., Adnan M., Amanullah (2019). Suppressing Photorespiration for the Improvement in Photosynthesis and Crop Yields: A Review on the Role of S-Allantoin as a Nitrogen Source. J. Environ. Manag..

[91] Lima-Melo Y., Alencar V.T.C.B., Lobo A.K.M., Sousa R.H.V., Tikkanen M., Aro E.-M., Silveira J.A.G., Gollan P.J. (2019). Photoinhibition of Photosystem I Provides Oxidative Protection During Imbalanced Photosynthetic Electron Transport in *Arabidopsis thaliana*. Front. Plant Sci..

[92] Mehdi F., Liu X., Riaz Z., Javed U., Aman A., Galani S. (2023). Expression of Sucrose Metabolizing Enzymes in Different Sugarcane Varieties under Progressive Heat Stress. Front. Plant Sci..

[93] Sharma N., Thakur M., Suryakumar P., Mukherjee P., Raza A., Prakash C.S., Anand A. (2022). ‘Breathing Out’ under Heat Stress—Respiratory Control of Crop Yield under High Temperature. Agronomy.

[94] Salvucci M.E., Crafts-Brandner S.J. (2004). Inhibition of Photosynthesis by Heat Stress: The Activation State of Rubisco as a Limiting Factor in Photosynthesis. Physiol. Plant..

[95] Yu J., Yang Z., Jespersen D., Huang B. (2014). Photosynthesis and Protein Metabolism Associated with Elevated CO2-Mitigation of Heat Stress Damages in Tall Fescue. Environ. Exp. Bot..

[96] Ahammed G.J., Li X., Zhou J., Zhou Y.-H., Yu J.-Q., Ahammed G.J., Yu J.-Q. (2016). Role of Hormones in Plant Adaptation to Heat Stress. Plant Hormones Under Challenging Environmental Factors.

[97] Sharma L., Priya M., Kaushal N., Bhandhari K., Chaudhary S., Dhankher O.P., Prasad P.V.V., Siddique K.H.M., Nayyar H. (2020). Plant Growth-Regulating Molecules as Thermoprotectants: Functional Relevance and Prospects for Improving Heat Tolerance in Food Crops. J. Exp. Bot..

[98] Postiglione A.E., Muday G.K. (2020). The Role of ROS Homeostasis in ABA-Induced Guard Cell Signaling. Front. Plant Sci..

[99] Liu P., Yin B., Gu L., Zhang S., Ren J., Wang Y., Duan W., Zhen W. (2023). Heat Stress Affects Tassel Development and Reduces the Kernel Number of Summer Maize. Front. Plant Sci..

[100] Sun J., Wang H., Ren H., Zhao B., Zhang J., Ren B., Liu P. (2023). Maize (*Zea mays* L.) Responses to Heat Stress: Mechanisms That Disrupt the Development and Hormone Balance of Tassels and Pollen. J. Agron. Crop Sci..

[101] Zhang K., Zhang Y., Sun J., Meng J., Tao J. (2021). Deterioration of Orthodox Seeds during Ageing: Influencing Factors, Physiological Alterations and the Role of Reactive Oxygen Species. Plant Physiol. Biochem..

[102] Żur I., Dubas E., Krzewska M., Janowiak F., Hura K., Pociecha E., Bączek-Kwinta R., Płażek A. (2014). Antioxidant Activity and ROS Tolerance in Triticale (×Triticosecale Wittm.) Anthers Affect the Efficiency of Microspore Embryogenesis. Plant Cell Tissue Organ Cult..

[103] Wang H., Sun J., Ren H., Zhao B., Ren B., Zhang J., Zhang Z., Li Y., Chen Y., Kuzyakov Y. (2025). Inhibiting Reactive Oxygen Species Production Mitigates Endoplasmic Reticulum Damage in Florets of Developing Maize Ears under Heat Stress. Plant J..

[104] Chaudhry A., Chen Z., Gallavotti A. (2024). Hormonal Influence on Maize Inflorescence Development and Reproduction. Plant Reprod..

[105] Xie D.-L., Zheng X.-L., Zhou C.-Y., Kanwar M.K., Zhou J. (2022). Functions of Redox Signaling in Pollen Development and Stress Response. Antioxidants.

[106] Duan L., Mo Z., Fan Y., Li K., Yang M., Li D., Ke Y., Zhang Q., Wang F., Fan Y. (2022). Genome-Wide Identification and Expression Analysis of the bZIP Transcription Factor Family Genes in Response to Abiotic Stress in *Nicotiana tabacum* L.. BMC Genom..

[107] Xiao D., Jiang Y., Wang Z., Li X., Li H., Tang S., Zhang J., Xia M., Zhang M., Deng X. (2025). Genome-Wide Identification and Expression Analysis of the HSP90 Gene Family in Relation to Developmental and Abiotic Stress in Ginger (*Zingiber officinale* Roscoe). Plants.

[108] Arenas-M A., Castillo F.M., Godoy D., Canales J., Calderini D.F. (2021). Transcriptomic and Physiological Response of Durum Wheat Grain to Short-Term Heat Stress during Early Grain Filling. Plants.

[109] Kumar D., Chattopadhyay S. (2018). Glutathione Modulates the Expression of Heat Shock Proteins via the Transcription Factors BZIP10 and MYB21 in *Arabidopsis*. J. Exp. Bot..

[110] Bielach A., Hrtyan M., Tognetti V.B. (2017). Plants under Stress: Involvement of Auxin and Cytokinin. Int. J. Mol. Sci..

[111] Zwack P.J., Rashotte A.M. (2015). Interactions between Cytokinin Signalling and Abiotic Stress Responses. J. Exp. Bot..

[112] Castro-Camba R., Sánchez C., Vidal N., Vielba J.M. (2022). Interactions of Gibberellins with Phytohormones and Their Role in Stress Responses. Horticulturae.

[113] Barbosa N.C.S., Dornelas M.C. (2021). The Roles of Gibberellins and Cytokinins in Plant Phase Transitions. Trop. Plant Biol..

[114] Hussain S., Chang J., Li J., Chen L., Ahmad S., Song Z., Zhang B., Chen X. (2025). Multifunctional Role of Cytokinin in Horticultural Crops. Int. J. Mol. Sci..

[115] Samanta S., Roychoudhury A. (2025). Molecular Crosstalk of Jasmonate with Major Phytohormones and Plant Growth Regulators during Diverse Stress Responses. J. Plant Growth Regul..

[116] Niharika, Singh N.B., Singh A., Khare S., Yadav V., Bano C., Yadav R.K. (2021). Mitigating Strategies of Gibberellins in Various Environmental Cues and Their Crosstalk with Other Hormonal Pathways in Plants: A Review. Plant Mol. Biol. Rep..

[117] Sarwar R., Zhu K.M., Jiang T., Ding P., Gao Y., Tan X.L. (2023). DELLAs Directed Gibberellins Responses Orchestrate Crop Development: A Brief Review. Crop Sci..

[118] Tavallali V., Darvishzadeh M.D. (2025). Synergistic Effects of Fe Nanocomplex and Nitrophenolate-Based Biostimulant on Growth and Physiological Performance of Tomato Seedlings. BMC Plant Biol..

[119] Li Y., Yuan W., Peng J., Ju J., Ling P., Guo X., Yang J., Ma Q., Lin H., Li J. (2024). GhGASA14 regulates the flowering time of upland cotton in response to GA_3_. Plant Cell Rep..

[120] Singh D., Dhiman V.K., Pandey H., Dhiman V.K., Pandey D. (2022). Crosstalk between Salicylic Acid and Auxins, Cytokinins, and Gibberellins under Biotic Stress. Auxins, Cytokinins and Gibberellins Signaling in Plants.

[121] Kumar R.R., Goswami S., Rai G.K., Jain N., Singh P.K., Mishra D., Chaturvedi K.K., Kumar S., Singh B., Singh G.P. (2021). Protection from Terminal Heat Stress: A Trade-Off between Heat-Responsive Transcription Factors (HSFs) and Stress-Associated Genes (SAGs) under Changing Environment. Cereal Res. Commun..

[122] Bouteraa M.T., Ben Romdhane W., Baazaoui N., Alfaifi M.Y., Chouaibi Y., Ben Akacha B., Ben Hsouna A., Kačániová M., Ćavar Zeljković S., Garzoli S. (2023). GASA Proteins: Review of Their Functions in Plant Environmental Stress Tolerance. Plants.

[123] Khan M.I.R., Fatma M., Per T.S., Anjum N.A., Khan N.A. (2015). Salicylic Acid-Induced Abiotic Stress Tolerance and Underlying Mechanisms in Plants. Front. Plant Sci..

[124] Shah S.A., Arshad M., Aslam S. (2025). Comprehensive Review on the Role of Exogenous Phytohormones in Enhancing Temperature Stress Tolerance in Plants. J. Crop Health.

[125] Ahanger M.A., Ashraf M., Bajguz A., Ahmad P. (2018). Brassinosteroids Regulate Growth in Plants Under Stressful Environments and Crosstalk with Other Potential Phytohormones. J. Plant Growth Regul..

[126] Galvão V.C., Collani S., Horrer D., Schmid M. (2015). Gibberellic Acid Signaling Is Required for Ambient Temperature-Mediated Induction of Flowering in *Arabidopsis thaliana*. Plant J..

[127] Kosakivska I., Voytenko L., Vasyuk V., Shcherbatiuk M. (2024). ABA-Induced Alterations in Cytokinin Homeostasis of *Triticum aestivum* and *Triticum spelta* under Heat Stress. Plant Stress.

[128] Voytenko L., Kosakivska I. (2025). AUXINS as regulators of growth and development of cereal crops under abiotic stresses: A review. Adv. Biol. Earth Sci..

[129] Boussora F., Allam M., Guasmi F., Ferchichi A., Rutten T., Hansson M., Youssef H.M., Börner A. (2019). Spike developmental stages and ABA role in spikelet primordia abortion contribute to the final yield in barley (*Hordeum vulgare* L.). Bot. Stud..

[130] Iqbal H., Yaning C., Raza S.T., Karim S., Shareef M., Waqas M. (2025). From Lab to Field: Harnessing H_2_O_2_-Mediated Upregulation of Plant Capacities under Abiotic Stresses. Physiol. Plant..

[131] Waseem M., Hasan M.M., Hazzazi Y., Alharbi B.M., Ghani M.U., Ahmad P., Carriquí M. (2025). Potential Mechanisms for the Rapid Post-Drought Reversal of ABA-Induced Stomatal Closure by Melatonin, 5-Aminolevulinic Acid, and Brassinosteroids. Photosynthetica.

[132] Tao Z., Yan P., Zhang X., Wang D., Wang Y., Ma X., Yang Y., Liu X., Chang X., Sui P. (2022). Physiological Mechanism of Abscisic Acid-Induced Heat-Tolerance Responses to Cultivation Techniques in Wheat and Maize. Agronomy.

[133] Kosakivska I., Voytenko L., Vasyuk V., Shcherbatiuk M. (2023). Aba-Induced Changes in Cytokinin Homeostasis of Cereals Under Heat Stress.

[134] Kosakivska I.V., Vasyuk V.A., Voytenko L.V., Shcherbatiuk M.M. (2022). Changes in Hormonal Status of Winter Wheat (*Triticum aestivum* L.) and Spelt Wheat (*Triticum spelta* L.) after Heat Stress and in Recovery Period. Cereal Res. Commun..

[135] Zhao J., Wang J., Liu J., Zhang P., Kudoyarova G., Liu C.J., Zhang K. (2024). Spatially distributed cytokinins: Metabolism, signaling, and transport. Plant Commun..

[136] Yao L., Li S., Zhou N., Guo Y. (2025). The Mechanism of Seed Priming with Abscisic Acid for Enhancing Cuticle Deposition Under Drought Stress: Phenotypic and Transcriptomic Insights. Agriculture.

[137] Onyemaobi I.O. (2018). Genetic Analysis of Meiotic-Stage Water Stress Tolerance in Wheat (*Triticum aestivum* L.). Ph.D. Thesis.

[138] Kosakivska I.V., Voytenko L.V., Vasyuk V.A., Shcherbatiuk M.M. (2023). Abscisic Acid-Induced Response of *Triticum aestivum* and *T. spelta* Phytohormonal System to Moderate Soil Drought. Zemdirb.-Agric..

[139] Kosakivska I.V., Vasyuk V.A., Voytenko L.V., Shcherbatiuk M.M. (2023). The Effects of Moderate Soil Drought on Phytohormonal Balance of *Triticum aestivum* L. and *Triticum spelta* L. *Cereal Res*. Commun..

[140] Kosakivska I.V., Voytenko L.V., Vasyuk V.A., Shcherbatiuk M.M. (2022). Effect of Pre-Sowing Priming of Seeds with Exogenous Abscisic Acid on Endogenous Hormonal Balance of Spelt Wheat under Heat Stress. Zemdirb.-Agric..

[141] Han H., Tian Z., Fan Y., Cui Y., Cai J., Jiang D., Cao W., Dai T. (2015). Water-deficit treatment followed by re-watering stimulates seminal root growth associated with hormone balance and photosynthesis in wheat (*Triticum aestivum* L.) seedlings. Plant Growth Regul..

[142] Hudeček M., Nožková V., Plíhalová L., Plíhal O. (2023). Plant hormone cytokinin at the crossroads of stress priming and control of photosynthesis. Front. Plant Sci..

[143] Iqbal N., Khan N.A., Ferrante A., Trivellini A., Francini A., Khan M.I.R. (2017). Ethylene Role in Plant Growth, Development and Senescence: Interaction with Other Phytohormones. Front. Plant Sci..

[144] Poór P., Nawaz K., Gupta R., Ashfaque F., Khan M.I.R. (2022). Ethylene Involvement in the Regulation of Heat Stress Tolerance in Plants. Plant Cell Rep..

[145] Janaagal M., Sharma P., Kumari G., Gulia H., Suresh G., Tallapragada S., Devi S., Lakra N., Arya S.S., Pooja P. (2024). Revolutionizing High Temperature Stress Relief: Exploring the Latest Advances in Salicylic Acid Application. J. Crop Health.

[146] Rehman A., Khan I., Farooq M. (2024). Secondary Metabolites Mediated Reproductive Tolerance Under Heat Stress in Plants. J. Plant Growth Regul..

[147] Fahad S., Hussain S., Bano A., Saud S., Hassan S., Shan D., Khan F.A., Khan F., Chen Y., Wu C. (2015). Potential Role of Phytohormones and Plant Growth-Promoting Rhizobacteria in Abiotic Stresses: Consequences for Changing Environment. Environ. Sci. Pollut. Res..

[148] Rudolf J., Tomovicova L., Panzarova K., Fajkus J., Hejatko J., Skalak J. (2024). Epigenetics and plant hormone dynamics: A functional and methodological perspective. J. Exp. Bot..

[149] Lohani N., Singh M.B., Bhalla P.L. (2020). High Temperature Susceptibility of Sexual Reproduction in Crop Plants. J. Exp. Bot..

[150] Burgess A.J., Masclaux-Daubresse C., Strittmatter G., Weber A.P., Taylor S.H., Harbinson J., Yin X., Long S., Paul M.J., Westhoff P. (2023). Improving crop yield potential: Underlying biological processes and future prospects. Food Energy Secur..

[151] Resentini F., Orozco-Arroyo G., Cucinotta M., Mendes M.A. (2023). The Impact of Heat Stress in Plant Reproduction. Front. Plant Sci..

[152] Mehmood M., Tanveer N.A., Joyia F.A., Ullah I., Mohamed H.I. (2025). Effect of High Temperature on Pollen Grains and Yield in Economically Important Crops: A Review. Planta.

[153] Endo M., Tsuchiya T., Hamada K., Kawamura S., Yano K., Ohshima M., Higashitani A., Watanabe M., Kawagishi-Kobayashi M. (2009). High Temperatures Cause Male Sterility in Rice Plants with Transcriptional Alterations during Pollen Development. Plant Cell Physiol..

[154] Jagadish S.V.K., Pal M., Singh S.N. (2009). Response of Rice (*Oryza sativa* L.) to Increasing Temperature and Atmospheric CO_2_. Climate Change and Crops.

[155] Usman B., Nawaz G., Zhao N., Liu Y., Li R. (2020). Generation of High Yielding and Fragrant Rice (*Oryza sativa* L.) Lines by CRISPR/Cas9 Targeted Mutagenesis of Three Homoeologs of Cytochrome P450 Gene Family and OsBADH2 and Transcriptome and Proteome Profiling Revealed Changes Triggered by Mutations. Plants.

[156] Evans L.T. (1987). Short Day Induction of Inflorescence Initiation in Some Winter Wheat Varieties. Funct. Plant Biol..

[157] Shitsukawa N., Kinjo H., Takumi S., Murai K. (2009). Heterochronic Development of the Floret Meristem Determines Grain Number per Spikelet in Diploid, Tetraploid and Hexaploid Wheats. Ann. Bot..

[158] Wang H.M., Enns J.L., Nelson K.L., Brost J.M., Orr T.D., Ferrie A.M.R. (2019). Improving the Efficiency of Wheat Microspore Culture Methodology: Evaluation of Pretreatments, Gradients, and Epigenetic Chemicals. Plant Cell Tissue Organ Cult..

[159] Ugarte C., Calderini D.F., Slafer G.A. (2007). Grain Weight and Grain Number Responsiveness to Pre-Anthesis Temperature in Wheat, Barley and Triticale. Field Crops Res..

[160] Calderini D.F., Reynolds M.P. (2000). Changes in Grain Weight as a Consequence of De-Graining Treatments at Pre- and Post-Anthesis in Synthetic Hexaploid Lines of Wheat (*Triticum durum* × *T. tauschii*). Funct. Plant Biol..

[161] Kaur V., Behl R. (2010). Grain Yield in Wheat as Affected by Short Periods of High Temperature, Drought and Their Interaction during Pre- and Post-Anthesis Stages. Communications.

[162] Brenciiley W.E. (1920). The Development of the Flower and Grain of Barley. J. Inst. Brew..

[163] Jacquard C., Mazeyrat-Gourbeyre F., Devaux P., Boutilier K., Baillieul F., Clément C. (2009). Microspore Embryogenesis in Barley: Anther Pre-Treatment Stimulates Plant Defence Gene Expression. Planta.

[164] Abdalla Eltantawy A. (2016). Key Factors Involved in Stress-Induced Microspore Embryogenesis in Barley and Rapeseed: DNA Methylation, Arabinogalactan Proteins and Auxin.

[165] Oshino T., Abiko M., Saito R., Ichiishi E., Endo M., Kawagishi-Kobayashi M., Higashitani A. (2007). Premature Progression of Anther Early Developmental Programs Accompanied by Comprehensive Alterations in Transcription during High-Temperature Injury in Barley Plants. Mol. Genet. Genom..

[166] García G.A., Serrago R.A., Dreccer M.F., Miralles D.J. (2016). Post-Anthesis Warm Nights Reduce Grain Weight in Field-Grown Wheat and Barley. Field Crops Res..

[167] Hong S.-Y., Park J.-H., Cho S.-H., Yang M.-S., Park C.-M. (2011). Phenological Growth Stages of Brachypodium Distachyon: Codification and Description. Weed Res..

[168] Sharma A., Singh M.B., Bhalla P.L. (2015). Ultrastructure of Microsporogenesis and Microgametogenesis in Brachypodium Distachyon. Protoplasma.

[169] Lin J.-J., Dickinson D.B. (1984). Ability of Pollen to Germinate Prior to Anthesis and Effect of Desiccation on Germination 1. Plant Physiol..

[170] Peng Y., Zeng X., Houx III J.H., Boardman D.L., Li C., Fritschi F.B. (2016). Pre- and Post-Silking Carbohydrate Concentrations in Maize Ear-Leaves and Developing Ears in Response to Nitrogen Availability. Crop Sci..

[171] Amelework A., Laing M., Shimelis H. (2016). Evaluation of Effective Gametocides for Selective Induction of Male Sterility in Sorghum. Czech J. Genet. Plant Breed..

[172] Bartek M.S., Hodnett G.L., Burson B.L., Stelly D.M., Rooney W.L. (2012). Pollen Tube Growth after Intergeneric Pollinations of Iap-Homozygous Sorghum. Crop Sci..

[173] Laza H.E., Kaur-Kapoor H., Xin Z., Payton P.R., Chen J. (2022). Morphological Analysis and Stage Determination of Anther Development in Sorghum [*Sorghum bicolor* (L.) Moench]. Planta.

[174] Worland B., Robinson N., Jordan D., Schmidt S., Godwin I. (2017). Post-Anthesis Nitrate Uptake Is Critical to Yield and Grain Protein Content in *Sorghum bicolor*. J. Plant Physiol..

[175] Gupta S.K., Rai K.N., Singh P., Ameta V.L., Gupta S.K., Jayalekha A.K., Mahala R.S., Pareek S., Swami M.L., Verma Y.S. (2015). Seed Set Variability under High Temperatures during Flowering Period in Pearl Millet (*Pennisetum glaucum* L. (R.) Br.). Field Crops Res..

[176] Miao Y., Cao J., Huang L., Yu Y., Lin S. (2021). FLA14 Is Required for Pollen Development and Preventing Premature Pollen Germination under High Humidity in *Arabidopsis*. BMC Plant Biol..

[177] Osorio Zaldumbide E.E. (2020). Effect of High Temperature on Ovule Development in Field Pea (*Pisum sativum* L.). Ph.D. Dissertation.

[178] Jerca I.O., Cîmpeanu S.M., Teodorescu R.I., Drăghici E.M., Nițu O.A., Sannan S., Arshad A. (2024). A Comprehensive Assessment of the Morphological Development of Inflorescence, Yield Potential, and Growth Attributes of Summer-Grown, Greenhouse Cherry Tomatoes. Agronomy.

[179] Mattioli R., Biancucci M., El Shall A., Mosca L., Costantino P., Funck D., Trovato M. (2018). Proline Synthesis in Developing Microspores Is Required for Pollen Development and Fertility. BMC Plant Biol..

[180] Yang D., Wang Z., Huang X., Xu C. (2023). Molecular Regulation of Tomato Male Reproductive Development. aBIOTECH.

[181] Tran L.T., Sugimoto K., Kasozi M., Mitalo O.W., Ezura H. (2023). Pollination, Pollen Tube Growth, and Fertilization Independently Contribute to Fruit Set and Development in Tomato. Front. Plant Sci..

[182] Patterson B.D., Mutton L., Paull R.E., Nguyen V.Q. (1987). Tomato Pollen Development: Stages Sensitive to Chilling and a Natural Environment for the Selection of Resistant Genotypes. Plant Cell Environ..

[183] Farooq M., Rehman A., Wahid A., Siddique K.H.M. (2021). Physiology of Grain Development in Cereals. Handbook of Plant and Crop Physiology.

[184] Wang C., Yang X., Li G. (2021). Molecular Insights into Inflorescence Meristem Specification for Yield Potential in Cereal Crops. Int. J. Mol. Sci..

[185] Mohapatra P.K., Sahu B.B., Mohapatra P.K., Sahu B.B. (2022). Botany of Rice Plant. Panicle Architecture of Rice and Its Relationship with Grain Filling.

[186] Xie W., He P., Ma H., Huang X., Fan G., Yang H. (2023). Straw Mulching Combined with Phosphorus Fertilizer Increases Fertile Florets of Wheat by Enhancing Leaf Photosynthesis and Assimilate Utilization. Agronomy.

[187] Kosakivska I.V., Vedenicheva N.P., Babenko L.M., Voytenko L.V., Romanenko K.O., Vasyuk V.A. (2022). Exogenous Phytohormones in the Regulation of Growth and Development of Cereals under Abiotic Stresses. Mol. Biol. Rep..

[188] Mohapatra P.K., Sahu B.B., Mohapatra P.K., Sahu B.B. (2022). Ontogeny of Organ Development in Rice Plant. Panicle Architecture of Rice and its Relationship with Grain Filling.

[189] Liu Z. (2025). Mutant Analysis in a Small Weed Reveals the Blueprint of Floral Patterning. Dev. Biol..

[190] Bowman J.L., Moyroud E. (2024). Reflections on the ABC Model of Flower Development. Plant Cell.

[191] Pfannebecker K.C., Lange M., Rupp O., Becker A. (2016). An Evolutionary Framework for Carpel Developmental Control Genes. Mol. Biol. Evol..

[192] Cong D., Zhao X., Ni C., Li M., Han L., Cheng J., Liu H., Liu H., Yao D., Liu S. (2025). The SEPALLATA-like Gene HrSEP1 in Hippophae Rhamnoides Regulates Flower Development by Interacting with Other MADS-Box Subfamily Genes. Front. Plant Sci..

[193] Adhikari P.B., Kasahara R.D. (2024). An Overview on MADS Box Members in Plants: A Meta-Review. Int. J. Mol. Sci..

[194] Zhou L., Iqbal A., Yang M., Yang Y. (2025). Research Progress on Gene Regulation of Plant Floral Organogenesis. Genes.

[195] Magon G. (2023). The Regulation of Gene Expression in Grapevine Flowering Process. Ph.D. Thesis.

[196] Zhang Z., Hu M., Xu W., Wang Y., Huang K., Zhang C., Wen J. (2021). Understanding the molecular mechanism of anther development under abiotic stresses. Plant Mol. Biol..

[197] Zenda T., Wang N., Dong A., Zhou Y., Duan H. (2022). Reproductive-stage heat stress in cereals: Impact, plant responses and strategies for tolerance improvement. Int. J. Mol. Sci..

[198] Matsoukas I.G., Massiah A.J., Thomas B. (2012). Florigenic and Antiflorigenic Signaling in Plants. Plant Cell Physiol..

[199] Lymperopoulos P., Msanne J., Rabara R. (2018). Phytochrome and Phytohormones: Working in Tandem for Plant Growth and Development. Front. Plant Sci..

[200] Loka D.A., Oosterhuis D.M. (2016). Increased Night Temperatures during Cotton’s Early Reproductive Stage Affect Leaf Physiology and Flower Bud Carbohydrate Content Decreasing Flower Bud Retention. J. Agron. Crop Sci..

[201] Chiluwal A., Bheemanahalli R., Kanaganahalli V., Boyle D., Perumal R., Pokharel M., Oumarou H., Jagadish S.V.K. (2020). Deterioration of Ovary Plays a Key Role in Heat Stress-Induced Spikelet Sterility in Sorghum. Plant Cell Environ..

[202] Kaur I., Kathpalia R., Koul M. (2024). Understanding Megasporogenesis through Model Plants: Contemporary Evidence and Future Insights. Int. J. Dev. Biol..

[203] Diwan G., Rawte S., Jha Z., Jha Z., Verulkar S.B., Penna S. (2025). Haploids: Then and Now. Doubled Haploids: Technological Advances and Role in Crop Improvement.

[204] Wang X.T., Yuan C., Yuan T.T., Cui S.J. (2012). The *Arabidopsis* LFR Gene Is Required for the Formation of Anther Cell Layers and Normal Expression of Key Regulatory Genes. Mol. Plant.

[205] Ma H. (2005). Molecular genetic analyses of microsporogenesis and microgametogenesis in flowering plants. Annu. Rev. Plant Biol..

[206] Qian Z., Shi D., Zhang H., Li Z., Huang L., Yan X., Lin S. (2024). Transcription Factors and Their Regulatory Roles in the Male Gametophyte Development of Flowering Plants. Int. J. Mol. Sci..

[207] De Jaeger-Braet J., Schnittger A. (2024). Heating up Meiosis—Chromosome Recombination and Segregation under High Temperatures. Curr. Opin. Plant Biol..

[208] Boateng K.A. (2007). Studies on Arabidopsis Proteins Required for the Establishment and Release of Sister Chromatid Cohesion. Ph.D. Thesis.

[209] Lohani N., Singh M.B., Bhalla P.L. (2024). Heat-Triggered Transcriptional Reprogramming in Microspores Disrupts Progression of Pollen Development in *Brassica napus* L.. BioRxiv.

[210] Maroto M., Torvisco S.N., García-Merino C., Fernández-González R., Pericuesta E. (2025). Mechanisms of Hormonal, Genetic, and Temperature Regulation of Germ Cell Proliferation, Differentiation, and Death During Spermatogenesis. Biomolecules.

[211] Huang L., Pant J., Dell B., Bell R.W. (2000). Effects of Boron Deficiency on Anther Development and Floret Fertility in Wheat (*Triticum aestivum* L. ‘Wilgoyne’). Ann. Bot..

[212] Zhang Z., Li Y., Wu Y., Zheng X., Guo X., Sun W., Sun Z., Wang Z., Zhang Y. (2024). A Dynamic Regulation of Nitrogen on Floret Primordia Development in Wheat. Crop J..

[213] Rutley N., Twell D. (2015). A Decade of Pollen Transcriptomics. Plant Reprod..

[214] Basiri E., Jafari Marandi S., Arbabian S., Majd A., Malboobi M.A. (2021). Development of Male and Female Gametophytes and Embryogenesis in the *Arabidopsis thaliana*. Biologia.

[215] Grossniklaus U., Schneitz K. (1998). The Molecular and Genetic Basis of Ovule and Megagametophyte Development. Semin. Cell Dev. Biol..

[216] Sprunck S., Groß-Hardt R. (2011). Nuclear Behavior, Cell Polarity, and Cell Specification in the Female Gametophyte. Sex. Plant Reprod..

[217] Shi W., Yang J., Kumar R., Zhang X., Impa S.M., Xiao G., Jagadish S.V.K. (2022). Heat Stress During Gametogenesis Irreversibly Damages Female Reproductive Organ in Rice. Rice.

[218] Erfatpour M., MacLean D., Lahlali R., Jiang Y. (2024). Ovule and seed development of crop plants in response to climate change. Front. Sustain. Food Syst..

[219] Hedhly A. (2011). Sensitivity of Flowering Plant Gametophytes to Temperature Fluctuations. Environ. Exp. Bot..

[220] Afzal S., Chaudhary N., Singh N.K. (2021). Role of soluble sugars in metabolism and sensing under abiotic stress. Plant Growth Regulators: Signalling Under Stress Conditions.

[221] Kumar S., Thakur M., Mitra R., Basu S., Anand A. (2022). Sugar Metabolism during Pre- and Post-Fertilization Events in Plants under High Temperature Stress. Plant Cell Rep..

[222] Raja M.M., Vijayalakshmi G., Naik M.L., Basha P.O., Sergeant K., Hausman J.F., Khan P.S.S.V. (2019). Pollen Development and Function under Heat Stress: From Effects to Responses. Acta Physiol. Plant.

[223] Feng Y., Li Z., Kong X., Khan A., Ullah N., Zhang X. (2025). Plant Coping with Cold Stress: Molecular and Physiological Adaptive Mechanisms with Future Perspectives. Cells.

[224] Dos Santos T.B., Ribas A.F., de Souza S.G.H., Budzinski I.G.F., Domingues D.S. (2022). Physiological Responses to Drought, Salinity, and Heat Stress in Plants: A Review. Stresses.

[225] Kan Y., Mu X.-R., Gao J., Lin H.-X., Lin Y. (2023). The Molecular Basis of Heat Stress Responses in Plants. Mol. Plant.

[226] Somero G.N. (2020). The Cellular Stress Response and Temperature: Function, Regulation, and Evolution. J. Exp. Zool. A Ecol. Integr. Physiol..

[227] Wen J., Qin Z., Sun L., Zhang Y., Wang D., Peng H., Yao Y., Hu Z., Ni Z., Sun Q. (2023). Alternative Splicing of TaHSFA6e Modulates Heat Shock Protein–Mediated Translational Regulation in Response to Heat Stress in Wheat. New Phytol..

[228] Liu G., Wang H., Gao H., Yu S., Liu C., Wang Y., Sun Y., Zhang D. (2025). Alternative Splicing of Functional Genes in Plant Growth, Development, and Stress Responses. Int. J. Mol. Sci..

[229] Merret R., Carpentier M.C., Favory J.J., Picart C., Descombin J., Bousquet-Antonelli C., Tillard P., Lejay L., Deragon J.M., Charng Y.Y. (2017). Heat Shock Protein HSP101 Affects the Release of Ribosomal Protein mRNAs for Recovery after Heat Shock. Plant Physiol..

[230] Tian X., Qin Z., Zhao Y., Wen J., Lan T., Zhang L., Wang F., Qin D., Yu K., Zhao A. (2022). Stress Granule-Associated TaMBF1c Confers Thermotolerance through Regulating Specific mRNA Translation in Wheat (*Triticum aestivum*). New Phytol..

[231] Blombach F., Launay H., Snijders A.P., Zorraquino V., Wu H., de Koning B., Brouns S.J., Ettema T.J., Camilloni C., Cavalli A. (2014). Archaeal MBF1 binds to 30S and 70S ribosomes via its helix–turn–helix domain. Biochem. J..

[232] Mahmood T., Safdar W., Abbasi B.H., Naqvi S.M.S. (2010). An Overview on the Small Heat Shock Proteins. Afr. J. Biotechnol..

[233] Pirkkala L., Nykänen P., Sistonen L.E.A. (2001). Roles of the Heat Shock Transcription Factors in Regulation of the Heat Shock Response and Beyond. FASEB J..

[234] Gomez-Pastor R., Burchfiel E.T., Thiele D.J. (2018). Regulation of Heat Shock Transcription Factors and Their Roles in Physiology and Disease. Nat. Rev. Mol. Cell Biol..

[235] Kumar P., Paul D., Jhajhriya S., Kumar R., Dutta S., Siwach P., Das S. (2024). Understanding Heat-Shock Proteins’ Abundance and Pivotal Function under Multiple Abiotic Stresses. J. Plant Biochem. Biotechnol..

[236] Lu F., Feng B., Chen L., Qiu J., Wei X. (2025). How Does Rice Cope with High-Temperature Stress During Its Growth and Development, Especially at the Grain-Filling Stage?. Agronomy.

[237] Swindell W.R., Huebner M., Weber A.P. (2007). Transcriptional Profiling of *Arabidopsis* Heat Shock Proteins and Transcription Factors Reveals Extensive Overlap between Heat and Non-Heat Stress Response Pathways. BMC Genom..

[238] Yurina N.P. (2023). Heat shock proteins in plant protection from oxidative stress. Mol. Biol..

[239] Pérez-Salamó I., Papdi C., Rigó G., Zsigmond L., Vilela B., Lumbreras V., Nagy I., Horváth B., Domoki M., Darula Z. (2014). The heat shock factor A4A confers salt tolerance and is regulated by oxidative stress and the mitogen-activated protein kinases MPK3 and MPK6. Plant Physiol..

[240] Waters E.R., Lee G.J., Vierling E. (1996). Evolution, Structure and Function of the Small Heat Shock Proteins in Plants. J. Exp. Bot..

[241] Heinemann B., Künzler P., Eubel H., Braun H.-P., Hildebrandt T.M. (2021). Estimating the Number of Protein Molecules in a Plant Cell: Protein and Amino Acid Homeostasis during Drought. Plant Physiol..

[242] Kolesnichenko A.V., Zykova V.V., Grabelnych O.I., Sumina O.N., Pobezhimova T.P., Voinikov V.K. (2000). Screening of Mitochondrial Proteins in Winter Rye, Winter Wheat, Elymus and Maize with an Immunochemical Affinity to the Stress Protein 310 kD and Their Intramitochondrial Localization in Winter Wheat. J. Therm. Biol..

[243] Suzuki N. (2023). Fine tuning of ROS, redox and energy regulatory systems associated with the functions of chloroplasts and mitochondria in plants under heat stress. Int. J. Mol. Sci..

[244] Yadav M.R., Choudhary M., Singh J., Lal M.K., Jha P.K., Udawat P., Gupta N.K., Rajput V.D., Garg N.K., Maheshwari C. (2022). Impacts, tolerance, adaptation, and mitigation of heat stress on wheat under changing climates. Int. J. Mol. Sci..

[245] Nasr M.A., Dovbeshko G.I., Bearne S.L., El-Badri N., Matta C.F. (2019). Heat Shock Proteins in the “Hot” Mitochondrion: Identity and Putative Roles. BioEssays.

[246] Schmitt M., Neupert W., Langer T. (1996). The Molecular Chaperone Hsp78 Confers Compartment-Specific Thermotolerance to Mitochondria. J. Cell Biol..

[247] Webster J.M., Darling A.L., Uversky V.N., Blair L.J. (2019). Small Heat Shock Proteins, Big Impact on Protein Aggregation in Neurodegenerative Disease. Front. Pharmacol..

[248] Young T.E., Ling J., Geisler-Lee C.J., Tanguay R.L., Caldwell C., Gallie D.R. (2001). Developmental and Thermal Regulation of the Maize Heat Shock Protein, HSP101. Plant Physiol..

[249] Tiwari L.D., Kumar R., Sharma V., Sahu A.K., Sahu B., Naithani S.C., Grover A. (2021). Stress and Development Phenotyping of Hsp101 and Diverse Other Hsp Mutants of *Arabidopsis thaliana*. J. Plant Biochem. Biotechnol..

[250] Haq N.U., Ammar M., Bano A., Luthe D.S., Heckathorn S.A., Shakeel S.N. (2013). Molecular Characterization of Chenopodium Album Chloroplast Small Heat Shock Protein and Its Expression in Response to Different Abiotic Stresses. Plant Mol. Biol. Rep..

[251] Khan Z., Shahwar D., Roychowdhury R., Choudhury S., Hasanuzzaman M., Srivastava S. (2020). Role of Heat Shock Proteins (HSPs) and Heat Stress Tolerance in Crop Plants. Sustainable Agriculture in the Era of Climate Change.

[252] Hu Y., Zhang T., Liu Y., Li Y., Wang M., Zhu B., Liao D., Yun T., Huang W., Zhang W. (2021). Pumpkin (Cucurbita Moschata) HSP20 Gene Family Identification and Expression Under Heat Stress. Front. Genet..

[253] Hu S., Ding Y., Zhu C. (2020). Sensitivity and Responses of Chloroplasts to Heat Stress in Plants. Front. Plant Sci..

[254] Momcilovic I., Ristic Z. (2004). Localization and Abundance of Chloroplast Protein Synthesis Elongation Factor (EF-Tu) and Heat Stability of Chloroplast Stromal Proteins in Maize. Plant Sci..

[255] Razzaq M.K., Rani R., Xing G., Xu Y., Raza G., Aleem M., Iqbal S., Arif M., Mukhtar Z., Nguyen H.T. (2023). Genome-Wide Identification and Analysis of the Hsp40/J-Protein Family Reveals Its Role in Soybean (*Glycine max*) Growth and Development. Genes.

[256] Haq S., Khan A., Ali M., Khattak A.M., Gai W.-X., Zhang H.-X., Wei A.-M., Gong Z.-H. (2019). Heat Shock Proteins: Dynamic Biomolecules to Counter Plant Biotic and Abiotic Stresses. Int. J. Mol. Sci..

[257] DeRocher A.E., Vierling E. (1994). Developmental Control of Small Heat Shock Protein Expression during Pea Seed Maturation. Plant J..

[258] Charfeddine S., Saïdi M.N., Charfeddine M., Gargouri-Bouzid R. (2015). Genome-Wide Identification and Expression Profiling of the Late Embryogenesis Abundant Genes in Potato with Emphasis on Dehydrins. Mol. Biol. Rep..

[259] Bies-Ethève N., Gaubier-Comella P., Debures A., Lasserre E., Jobet E., Raynal M., Cooke R., Delseny M. (2008). Inventory, Evolution and Expression Profiling Diversity of the LEA (Late Embryogenesis Abundant) Protein Gene Family in *Arabidopsis thaliana*. Plant Mol. Biol..

[260] Garcia-Bañuelos M.L., Gardea A.A., Winzerling J.J., Vazquez-Moreno L. (2009). Characterization of a Midwinter-Expressed Dehydrin (DHN) Gene from Apple Trees (Malus Domestica). Plant Mol. Biol. Rep..

[261] Ismail A.M., Hall A.E., Close T.J. (1999). Allelic Variation of a Dehydrin Gene Cosegregates with Chilling Tolerance during Seedling Emergence. Proc. Natl. Acad. Sci. USA.

[262] Ganguly M., Datta K., Roychoudhury A., Gayen D., Sengupta D.N., Datta S.K. (2012). Overexpression of Rab16A Gene in Indica Rice Variety for Generating Enhanced Salt Tolerance. Plant Signal. Behav..

[263] Rorat T. (2006). Plant Dehydrins—Tissue Location, Structure and Function. Cell Mol. Biol. Lett..

[264] Campbell S.A., Close T.J. (1997). Dehydrins: Genes, Proteins, and Associations with Phenotypic Traits. New Phytol..

[265] Zaidi I., Hanin M., Saidi M.N., Soltani N., Brini F. (2024). The Barley Dehydrin 4 and Stress Tolerance: From Gene to Function. Environ. Exp. Bot..

[266] Soltani N., Zaidi I., Saidi M.N., Brini F. (2025). Group 1 LEA Proteins in Durum Wheat: Evolution, Expression, and Roles in Abiotic Stress Tolerance. Plants.

[267] Lee D.-G., Ahsan N., Lee S.-H., Kang K.Y., Bahk J.D., Lee I.-J., Lee B.-H. (2007). A Proteomic Approach in Analyzing Heat-Responsive Proteins in Rice Leaves. Proteomics.

[268] Wahid A., Close T.J. (2007). Expression of Dehydrins under Heat Stress and Their Relationship with Water Relations of Sugarcane Leaves. Biol. Plant.

[269] Marcon C., Schützenmeister A., Schütz W., Madlung J., Piepho H.P., Hochholdinger F. (2010). Nonadditive Protein Accumulation Patterns in Maize (*Zea mays* L.) Hybrids during Embryo Development. J. Proteome Res..

[270] Jia J., Zhou J., Shi W., Cao X., Luo J., Polle A., Luo Z.-B. (2017). Comparative Transcriptomic Analysis Reveals the Roles of Overlapping Heat-/Drought-Responsive Genes in Poplars Exposed to High Temperature and Drought. Sci. Rep..

[271] Singh S., Praveen A., Dudha N., Bhadrecha P. (2024). Integrating Physiological and Multi-Omics Methods to Elucidate Heat Stress Tolerance for Sustainable Rice Production. Physiol. Mol. Biol. Plants.

[272] Liu X.-H., Lyu Y.-S., Yang W., Yang Z.-T., Lu S.-J., Liu J.-X. (2020). A Membrane-Associated NAC Transcription Factor OsNTL3 Is Involved in Thermotolerance in Rice. Plant Biotechnol. J..

[273] Shu Y., Zhou Y., Mu K., Hu H., Chen M., He Q., Huang S., Ma H., Yu X. (2020). A Transcriptomic Analysis Reveals Soybean Seed Pre-Harvest Deterioration Resistance Pathways under High Temperature and Humidity Stress. Genome.

[274] Fang S., Cammarano D., Zhou G., Tan K., Ren S. (2015). Effects of Increased Day and Night Temperature with Supplemental Infrared Heating on Winter Wheat Growth in North China. Eur. J. Agron..

[275] Ali I., Ullah S., Iqbal A., Quan Z., Liang H., Ahmad S., Muhammad I., Amanullah, Imran, Guo Z. (2021). Combined Application of Biochar and Nitrogen Fertilizer Promotes the Activity of Starch Metabolism Enzymes and the Expression of Related Genes in Rice in a Dual Cropping System. BMC Plant Biol..

[276] Kino R.I., Pellny T.K., Mitchell R.A.C., Gonzalez-Uriarte A., Tosi P. (2020). High Post-Anthesis Temperature Effects on Bread Wheat (*Triticum aestivum* L.) Grain Transcriptome during Early Grain-Filling. BMC Plant Biol..

[277] Zhang H., Zhang L., Lv H., Yu Z., Zhang D., Zhu W. (2014). Identification of changes in *Triticum aestivum* L. leaf proteome in response to drought stress by 2D-PAGE and MALDI-TOF/TOF mass spectrometry. Acta Physiol. Plant..

[278] Zhao Y., Zhao J., Hu M., Sun L., Liu Q., Zhang Y., Li Q., Wang P., Ma W., Li H. (2023). Transcriptome and Proteome Analysis Revealed the Influence of High-Molecular-Weight Glutenin Subunits (HMW-GSs) Deficiency on Expression of Storage Substances and the Potential Regulatory Mechanism of HMW-GSs. Foods.

[279] Rienth M., Torregrosa L., Luchaire N., Chatbanyong R., Lecourieux D., Kelly M.T., Romieu C. (2014). Day and Night Heat Stress Trigger Different Transcriptomic Responses in Green and Ripening Grapevine (Vitis Vinifera) Fruit. BMC Plant Biol..

[280] Balfagón D., Zandalinas S.I., Mittler R., Gómez-Cadenas A. (2020). High temperatures modify plant responses to abiotic stress conditions. Physiol. Plant..

[281] Riaz A., Thomas J., Ali H.H., Zaheer M.S., Ahmad N., Pereira A. (2024). High night temperature stress on rice (*Oryza sativa*)—Insights from phenomics to physiology: A review. Funct. Plant Biol..

[282] Teng Z., Chen Y., Meng S., Duan M., Zhang J., Ye N. (2023). Environmental Stimuli: A Major Challenge during Grain Filling in Cereals. Int. J. Mol. Sci..

[283] Seni S., Kaur S., Malik P., Yadav I.S., Sirohi P., Chauhan H., Kaur A., Chhuneja P. (2021). Transcriptome based identification and validation of heat stress transcription factors in wheat progenitor species *Aegilops speltoides*. Sci. Rep..

[284] Tomás D., Viegas W., Silva M. (2022). Grain Transcriptome Dynamics Induced by Heat in Commercial and Traditional Bread Wheat Genotypes. Front. Plant Sci..

[285] Song Y., Zhu Z., Liu K., Zhao Y., Nie Z., Zhang L., Muhammad Fahim A., Yang X. (2023). Comparative Transcriptome Analysis Reveals Differential Gene Expression Pattern Associated with Heat Tolerance in Pepper (*Capsicum annuum* L.). Horticulturae.

[286] Ni Z., Li H., Zhao Y., Peng H., Hu Z., Xin M., Sun Q. (2018). Genetic Improvement of Heat Tolerance in Wheat: Recent Progress in Understanding the Underlying Molecular Mechanisms. Crop J..

[287] Sharma E., Borah P., Kaur A., Bhatnagar A., Mohapatra T., Kapoor S., Khurana J.P. (2021). A Comprehensive Transcriptome Analysis of Contrasting Rice Cultivars Highlights the Role of Auxin and ABA Responsive Genes in Heat Stress Response. Genomics.

[288] Gul R.M.S., Rauf S., Ortiz R., Waqas Khalid M., Kaya Y. (2024). Understanding Abscisic Acid-Mediated Stress Signaling to Affect Rice Development under Stress. Front. Sustain. Food Syst..

[289] Guo M., Liu J.H., Ma X., Luo D.X., Gong Z.H., Lu M.H. (2016). The Plant Heat Stress Transcription Factors (HSFs): Structure, Regulation, and Function in Response to Abiotic Stresses. Front. Plant Sci..

[290] Ma W., Wang X., Gu C., Lu Z., Ma R., Wang X., Lu Y., Cai K., Tang Z., Zhou Z. (2025). Rice heat stress response: Physiological changes and molecular regulatory network research progress. Plants.

[291] Sharma N., Sharma L., Onkarappa D., Yogendra K., Bose J., Sharma R.A. (2024). Molecular Basis and Engineering Strategies for Transcription Factor-Mediated Reproductive-Stage Heat Tolerance in Crop Plants. Agronomy.

[292] Lamba K., Kumar M., Singh V., Chaudhary L., Gupta V. (2025). Transcriptome Analysis for Heat Stress Related Genes in Wheat Genotype WH-730. Cereal Res. Commun..

[293] Chaudhary C., Sharma N., Khurana P. (2021). Decoding the wheat awn transcriptome and overexpressing Ta Rca1β in rice for heat stress tolerance. Plant Mol. Biol..

[294] Talarico E., Zambelli A., Araniti F., Greco E., Chiappetta A., Bruno L. (2024). Unravelling the Epigenetic Code: DNA Methylation in Plants and Its Role in Stress Response. Epigenomes.

[295] Sharma M., Sidhu A.K., Samota M.K., Gupta M., Koli P., Choudhary M. (2023). Post-Translational Modifications in Histones and Their Role in Abiotic Stress Tolerance in Plants. Proteomes.

[296] Ramakrishnan M., Zhang Z., Mullasseri S., Kalendar R., Ahmad Z., Sharma A., Liu G., Zhou M., Wei Q. (2022). Epigenetic Stress Memory: A New Approach to Study Cold and Heat Stress Responses in Plants. Front. Plant Sci..

[297] Workman J.L., Kingston R.E. (1998). Alteration of Nucleosome Structure as a Mechanism of Transcriptional Regulation. Annu. Rev. Biochem..

[298] Luo M., Liu X., Singh P., Cui Y., Zimmerli L., Wu K. (2012). Chromatin Modifications and Remodeling in Plant Abiotic Stress Responses. Biochim. Et Biophys. Acta (BBA)—Gene Regul. Mech..

[299] Bure I.V., Nemtsova M.V., Kuznetsova E.B. (2022). Histone Modifications and Non-Coding RNAs: Mutual Epigenetic Regulation and Role in Pathogenesis. Int. J. Mol. Sci..

[300] Saha C., Saha S., Bhattacharyya N.P. (2025). LncRNAOmics: A Comprehensive Review of Long Non-Coding RNAs in Plants. Genes.

[301] Jin Q., Chachar M., Ali A., Chachar Z., Zhang P., Riaz A., Ahmed N., Chachar S. (2024). Epigenetic Regulation for Heat Stress Adaptation in Plants: New Horizons for Crop Improvement under Climate Change. Agronomy.

[302] Shanker A.K., Bhanu D., Maheswari M. (2020). Epigenetics and Transgenerational Memory in Plants under Heat Stress. Plant Physiol. Rep..

[303] Vaschetto L.M. (2024). DNA Methylation, Histone Modifications, and Non-coding RNA Pathways. Epigenetics in Crop Improvement: Safeguarding Food Security in an Ever-Changing Climate.

[304] Erdmann R.M., Picard C.L. (2020). RNA-directed DNA methylation. PLoS Genet..

[305] Ali S., Tang Y. (2025). Noncoding RNA-Mediated Regulation of DNA Methylation: Insights into Plant Epigenetic Mechanisms. J. Plant Growth Regul..

[306] Li Z., Gao Y., Zhang Y., Lin C., Gong D., Guan Y., Hu J. (2018). Reactive Oxygen Species and Gibberellin Acid Mutual Induction to Regulate Tobacco Seed Germination. Front. Plant Sci..

[307] Zdunek-Zastocka E., Grabowska A. (2019). The Interplay of *PsABAUGT1* with Other Abscisic Acid Metabolic Genes in the Regulation of ABA Homeostasis during the Development of Pea Seeds and Germination in the Presence of H_2_O_2_. Plant Sci..

[308] Sakai Y., Suriyasak C., Inoue M., Hamaoka N., Ishibashi Y. (2022). Heat stress during grain filling regulates seed germination through alterations of DNA methylation in barley (*Hordeum vulgare* L.). Plant Mol. Biol..

[309] He S., Zhang Y., Wang J., Wang Y., Ji F., Sun L., Zhang G., Hao F. (2022). H3K4me2, H4K5ac and DNA Methylation Function in Short- and Long-Term Heat Stress Responses through Affecting the Expression of the Stress-Related Genes in *G. Hirsutum*. Environ. Exp. Bot..

[310] Benamar K., Dehbi I., Radi M., Ez-Zouggari R., Fikri Benbrahim K., Jiang Y., Lahlali R. (2025). Epigenetics for Combating Heat Stress in Plants: Updated Methods and Current Achievements. Epigenetics for Climate-Smart and Sustainable Agriculture.

[311] Gan L., Wei Z., Yang Z., Li F., Wang Z. (2021). Updated Mechanisms of GCN5—The Monkey King of the Plant Kingdom in Plant Development and Resistance to Abiotic Stresses. Cells.

[312] Tahir M.S., Tian L. (2021). HD2-Type Histone Deacetylases: Unique Regulators of Plant Development and Stress Responses. Plant Cell Rep..

[313] Zhao J., Lu Z., Wang L., Jin B. (2021). Plant Responses to Heat Stress: Physiology, Transcription, Noncoding RNAs, and Epigenetics. Int. J. Mol. Sci..

[314] Margalha L., Elias A., Belda-Palazón B., Peixoto B., Confraria A., Baena-González E. (2023). HOS1 Promotes Plant Tolerance to Low-Energy Stress via the SnRK1 Protein Kinase. Plant J..

[315] Fernández-Jiménez N., Martinez-Garcia M., Varas J., Gil-Dones F., Santos J.L., Pradillo M. (2023). The Scaffold Nucleoporins SAR1 and SAR3 Are Essential for Proper Meiotic Progression in *Arabidopsis thaliana*. Front. Cell Dev. Biol..

